# Hematological and biochemical responses to three doses of caffeine during the CrossFit® workout: a randomized, double-blind, crossover trial

**DOI:** 10.1080/15502783.2026.2740805

**Published:** 2026-10-23

**Authors:** Natalia Główka, Tomasz Podgórski, Jakub Malik, Paulina M. Nowaczyk, Krzysztof Durkalec-Michalski

**Affiliations:** a Poznan University of Physical Education, Department of Sports Dietetics, Poznań, Poland; b Poznan University of Physical Education, Department of Biochemistry, Poznań, Poland; c Poznan University of Physical Education, Department of Sports Psychology, Poznań, Poland; d Polish Society of Nutritional Sciences, Warsaw, Poland

**Keywords:** High-intensity functional training, glucose metabolism, ergogenic aids, mineral homeostasis

## Abstract

**Background:**

This study investigated the effects of three doses of caffeine (CAF) on hematological and biochemical responses during the CrossFit® workout, a protocol known to induce significant physiological fluctuations.

**Methods:**

In a randomized, double-blind, placebo (*PLA*)-controlled crossover trial, 26 moderately trained CrossFit® athletes received acute doses of 3, 6, or 9 mg_CAF_/kg_body mass (BM)_, or a PLA, 70 min before a 17-min CrossFit® workout.

**Results:**

While exercise was the predominant driver of hematological changes, with no CAF-specific modulation of blood cell indices, significant treatment × time point interactions were observed solely for glucose concentration. Specifically, *MEDIUM* (6 mg_CAF_/kg_BM_) and *HIGH* (9 mg_CAF_/kg_BM_) CAF doses produced significantly greater post-exercise glucose elevations compared to baseline and *PLA* visits. Phosphates concentrations increased over time, particularly post-exercise, with no treatment effect retained after excluding baseline visit in the sensitivity analysis. Calcium level dynamics were influenced by temporal factors rather than CAF, and magnesium concentration remained stable.

**Conclusions:**

These findings suggest that acute CAF ingestion selectively influences post-exercise glucose responses during the CrossFit^®^ workout, whereas hematological variables and most biochemical markers remain unaffected. Significant post-exercise glucose elevations were observed following the 6 and 9 mg_CAF_/kg_BM_ conditions compared with baseline and *PLA visits*, while serum mineral concentrations remained largely stable across treatments.

**Clinical trials registration:**

NCT03822663

## 1. Introduction

Caffeine (CAF) is one of the most widely consumed dietary bioactive methylxanthines. While its performance-enhancing effects have been extensively documented across endurance, strength, and high-intensity exercise domains [1,2], recent interest has expanded toward its role in modulating systemic physiological responses to acute metabolic stress. Its primary mechanisms include competitive antagonism of adenosine receptors, resulting in reduced perception of effort, increased alertness and improved cognitive processing during exercise [1,3]. Additional physiological actions such as enhanced excitation-contraction coupling, increased sarcoplasmic calcium (Ca) ions release and altered substrate utilization may also contribute to improved exercise performance [4]. Although acute ingestion of 3–6 mg_CAF_/kg_body mass (BM)_ is typically recommended for ergogenic effects, substantial inter-individual variability has been observed, influenced by genetics, habitual CAF intake and training status [1,5,6]. Accordingly, three CAF doses representing the lower (3 mg/kg_BM_), commonly recommended (6 mg/kg_BM_), and upper (9 mg/kg_BM_) range of acute supplementation used in sports nutrition research were included. While these doses have been extensively investigated in relation to exercise performance, considerably less is known about whether increasing CAF doses differentially influence hematological and biochemical responses during high-intensity functional training. Addressing this knowledge gap may improve understanding of the physiological effects accompanying CAF supplementation beyond its established ergogenic actions.

High-intensity functional training (HIFT), frequently epitomized by the CrossFit® methodology, comprises multimodal, functional tasks executed at high intensity and imposes considerable cardiovascular, neuromuscular, and metabolic demands [7,8]. Acute CrossFit®-based HIFT sessions elicit pronounced perturbations in hematological and biochemical markers, mainly pronounced in exercise-induced leukocytosis, shifts in leukocyte subpopulations, hemoconcentration-related changes in hemoglobin (HGB) concentration and hematocrit (HCT) value, as well as alterations in glucose (Glu) concentrations [7,9,10]. Nevertheless, specific electrolyte concentration data following acute CrossFit® sessions is limited in the literature.

Given that CrossFit® combines resistance-type muscular loading with high metabolic stress, it represents a model in which both hematological and biochemical markers undergo rapid and meaningful physiological fluctuations reflecting the body's effort to maintain systemic homeostasis under extreme strain. In addition to its central effects, CAF acts as a potent bioactive modulator of several systemic responses relevant to these markers. Studies have shown that CAF can increase circulating Glu during or after exercise, likely through enhanced hepatic Glu output through gluconeogenesis and glycogenolysis processes, increased cortisol concentration and catecholamine-mediated metabolic stimulation [4,7]. Moreover, CAF intake has been associated with transient changes in mineral balance, including increased urinary Ca excretion. Although there are associations between coffee/CAF consumption and increased urinary Ca excretion, discussions on how physical activity influences laboratory biomarkers and fluid-electrolyte dynamics during exercise, specific evidence for reductions in serum or intracellular magnesium (Mg) concentrations and detailed phosphates (P) handling mechanisms related to CAF during exercise are still limited in the literature [11,12]. Moreover, it is demonstrated that hemoconcentration (via fluid removal) is associated with acute increases in HGB, HCT, and platelets (PLT), establishing the mechanistic plausibility [13], while CAF intake, as a dietary bioactive compound, increases sympathetic nerve activity and may alter exercise-related inflammatory or immune markers by modulating leukocyte-activity factors and the systemic response to oxidative stress [14,15].

In summary, these findings suggest that CAF, as a potent bioactive compound, has the potential to modulate both hematological and biochemical outcomes during high-intensity exercise. Recent findings from our previous study [16] demonstrated that CAF supplementation induces dose-dependent, task-specific changes in CrossFit® performance, reaction time and postural stability, with 6 mg_CAF_/kg_BM_ producing the most practically meaningful improvements. In another analysis involving the same athletes [17], we reported dose- and time-dependent alterations in CAF and paraxanthine concentrations in serum and saliva, although no changes in CYP1A2 enzymatic activity were observed. These results highlight that CAF elicits measurable ergogenic and metabolic responses under the same CrossFit® protocol; however, its influence on acute hematological and biochemical profiles remains unexplored within this controlled supplementation–exercise model. Therefore, the aim of this study was to examine whether acute ingestion of three different CAF doses (3, 6, and 9 mg_CAF_/kg_BM_) modulates hematological indices (white blood cells (WBC) count and differential, red blood cells (RBC)-related markers, HGB, HCT, and PLT indices), and biochemical markers (Glu, Ca, Mg, and P) during a standardized CrossFit®-specific workout protocol providing insight into how this bioactive component influences the physiological profile of athletes under high-intensity conditions. We hypothesized that different doses of CAF would differentially affect selected hematological and biochemical variables, reflecting both its pharmacological effects and the physiological demands of CrossFit®.

## 2. Materials and methods

### 2.1. Study design, protocol, and visits

This study utilized a randomized, double-blind, placebo (*PLA*)-controlled crossover design, where participants were given three different doses of CAF (3, 6, and 9 mg_CAF_/kg_BM_; labeled as *LOW*, *MEDIUM*, and *HIGH*, respectively) or a *PLA* on separate occasions. The study involved five laboratory visits (T_0_–T_4_). Initially, participants were familiarized with the study protocol, followed by the first visit (T_0_; baseline (*BASE*), without any supplementation). The *BASE* visit was included as a non-supplemented reference condition to characterize the physiological response to the standardized CrossFit® workout in the absence of supplementation. Including *BASE* in the repeated-measures model enabled comparison of the exercise response without supplementation with the subsequent *PLA* and CAF conditions within the same participants. Following the familiarization session, participants were stratified according to their performance in the *Fight Gone Bad* (FGB) workout (the number of repetitions). Within each stratum, the order of the four experimental conditions (*PLA*, *LOW*, *MEDIUM*, and *HIGH*) was determined using a computer-generated randomization sequence. The randomization sequence was generated by an independent researcher who was not involved in participant recruitment, data collection, or outcome assessment, thereby ensuring allocation concealment throughout the study. The main protocol included four visits (T_1_–T_4_), all conducted at the same time of day for each participant, three hours after a standardized meal (carbohydrates: 2 g/kg_BM_, proteins: 25 g, water: 7 mL/kg_BM_) [18,19]. Participants were instructed to avoid consuming any CAF-containing products for 24 h prior to each visit. Compliance with the 24-h CAF abstinence requirement was verified before each experimental session by reviewing participants' dietary records and through an interview conducted by a registered dietitian. Each visit involved the 17-min CrossFit® workout protocol and the collection of blood samples. A 7-day washout period was selected because it greatly exceeds the elimination time of CAF and has been widely used in previous crossover studies to minimize residual effects [20].

### 2.2. Participants

A total of 26 moderately trained CrossFit® athletes (10 females and 16 males) completed the entire study and were included in the analyses (Figure 1, Table 1). Male and female participants were analyzed together because the primary objective of the study was to evaluate the overall physiological responses to acute CAF supplementation during the CrossFit® workout. The study was not powered to detect sex-specific differences; therefore, sex-stratified analyses were not performed. As CrossFit® is defined as a strength and conditioning system built on constantly varied, functional movements executed at high intensity, all participants were recruited from CrossFit® clubs in Poznań, Poland. To be eligible, participants needed to be in good health, hold a valid medical certificate confirming their ability to participate in sports, have a minimum of 2 years of CrossFit® experience, train regularly at least 4 times per week, and regularly consume CAF-containing products (defined as more than two 240 mL cups of coffee per day). Exclusion criteria included any current injuries, health contraindications, general illness, refusal to comply with the study guidelines, smoking, and inability to maintain a 24-h CAF abstinence before each visit. The study was conducted between July 2021 and December 2022 at the Department of Sports Dietetics, Poznan University of Physical Education, Poland. Ethical approval was granted by the Bioethics Committee at Poznań University of Medical Sciences (reference number 293/17, May 11, 2017), and the trial was prospectively registered at ClinicalTrials.gov (NCT03822663, registration date: January 30, 2019). All participants provided written informed consent, and the procedures followed the ethical standards set forth in the 2013 Helsinki Declaration. A detailed visual description can be seen in Figure 1 (flow diagram).

**Table 1.** Baseline characteristics of the studied group (*n* = 26).

Baseline anthropometric and CAF intake characteristics	Mean ± SD
Age [years]	35.4 ± 6.5
Body mass [kg]	77.4 ± 16.6
Height [cm]	175 ± 9
Habitual CAF intake [mg/day]^a^	231 ± 121
‍**Baseline biochemical characteristics**	
‍AlAT [U/L]	30.24 ± 10.62
‍AspAT [U/L]	37.80 ± 14.86
‍CK [U/L]	417.37 ± 444.17
‍CREA [µmol/L]	95.83 ± 17.21
‍LDH [U/L]	454.94 ± 87.12
‍UREA [mmol/L]	7.03 ± 1.78

AlAT–alanine aminotransferase, AspAT–aspartate aminotransferase, CK–creatine kinase, CREA–creatinine, LDH–lactate dehydrogenase, *n*–number of participants, SD–standard deviation, UREA–urea.

^a^ Habitual daily intake, not including the day of the study and the 24 h preceding the visit, when participants were required not to consume any CAF sources.

**Figure 1.**
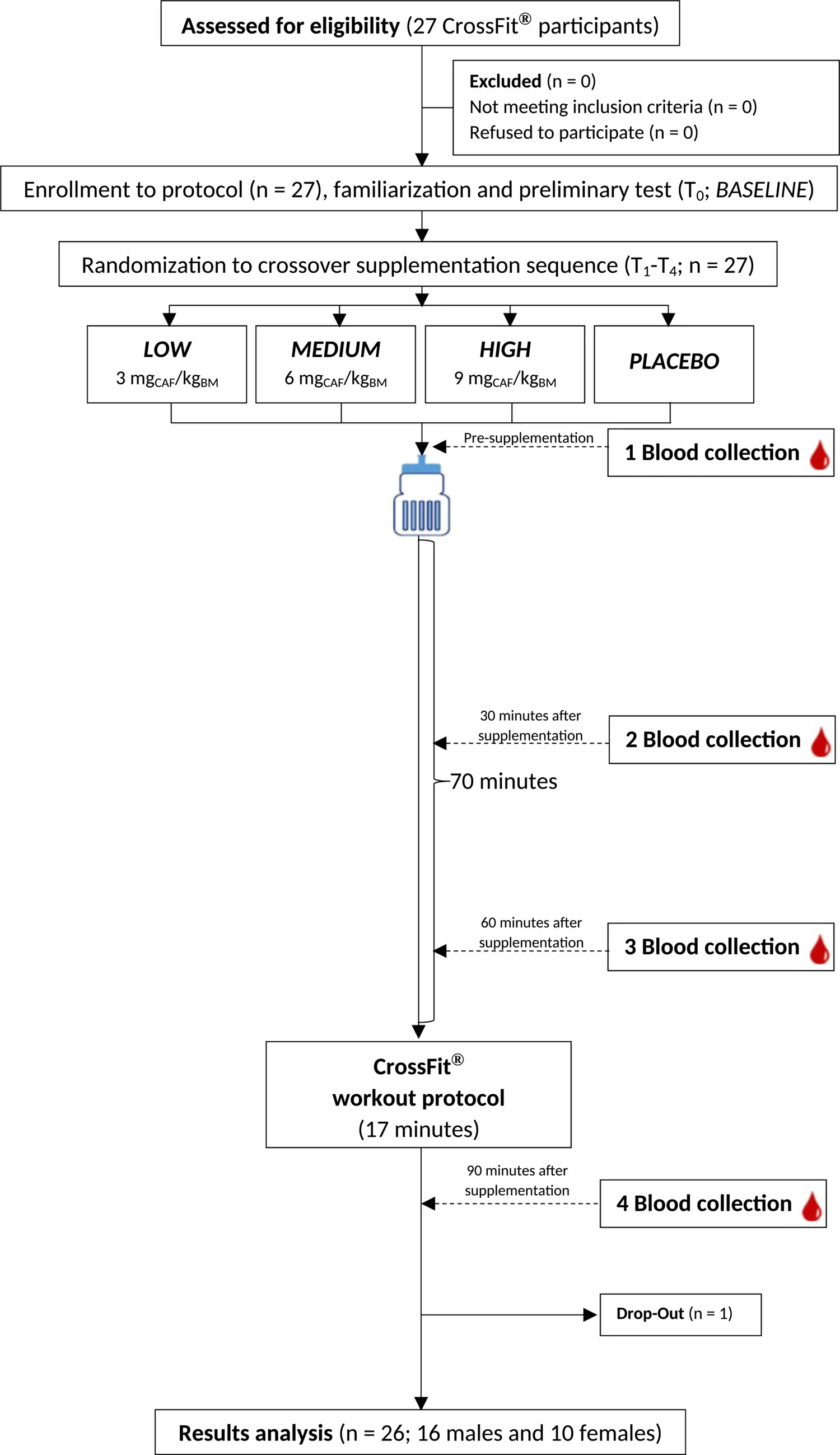
Flow chart of the study design.

### 2.3. Procedures

#### 2.3.1. Supplementation

In the experimental protocol, each participant received an acute dose of CAF [3 mg_CAF_/kg_BM_ (*LOW*), 6 mg_CAF_/kg_BM_ (*MEDIUM*), or 9 mg_CAF_/kg_BM_ (*HIGH*)] or *PLA* in a randomized crossover design. The CAF doses (pure pharmaceutical-grade CAF, OstroVit, Poland) and the PLA (a commercially available bitter flavoring without CAF) were dissolved in a mixture of 20 mL of orange juice and 80 mL of water. The PLA was formulated to mimic the bitter taste of CAF and was identical in appearance to the CAF beverages. Because only a small amount of flavoring was used, the PLA beverage did not differ meaningfully in energy content from the CAF beverages. These supplements were administered 70 min before the CrossFit® workout, following the first blood sample collection (Figure 1). The containers, labelled with unique codes, were prepared by a researcher not involved in the direct conduct of the study. To maintain double-blind conditions, neither the participants nor the researchers knew which supplementation (CAF or *PLA*) was being administered. Randomization details were kept confidential until the study was completed. Supplements were provided at a specific time before exercise, and adherence to consumption was closely monitored by the researchers.

#### 2.3.2. Exercise

The CrossFit®-specific FGB workout protocol was carried out over 17 min, following the method established in our previous studies [16,21,22]. Specifically, the protocol involved three rounds of five one-minute high-intensity exercises, with a one-minute rest interval between each round. This test is known for its high repeatability (ICC 0.9, SEM 6%) [22]. Prior to the commencement of the workout, participants completed a 20-min self-designed warm-up, which remained consistent for each individual across all study sessions. The findings related to the CrossFit® workout performance are reported in another publication [16].

#### 2.3.3. Blood collection and samples preparation

Capillary blood samples were collected at four time-points (TP) during each study session: 1) pre-supplementation (0′ TP), 2) 30 min after supplementation (30′ TP), 3) 60 min after supplementation (pre-exercise, 60′ TP), and 4) 3 min post-exercise (90 min after supplementation, 90′ TP). All samples were drawn by a trained medical professional while participants remained seated in an upright position. A total of 600 μL of blood was taken from the fingertip of the nondominant hand using a disposable Medlance® Red lancet-spike (HTL-STREFA, Łódź, Poland) with a blade depth of 1.5 mm and penetration depth of 2.0 mm. The capillary blood was each time collected into one Microvette® CB 300 K2E tube (Sarstedt, Nümbrect, Germany) for hematological and Microvette® CB 300 Z tube (Sarstedt, Nümbrect, Germany) for biochemical analyses.

#### 2.3.4. Hematological and biochemical analyses

At each TP analyses of WBC count and percentage distribution (lymphocytes, LYM; monocytes, MON; granulocytes, GRA) and hematological indices (red blood cells count, RBC; HGB; HCT; mean corpuscular volume, MCV; mean corpuscular hemoglobin mass, MCH; mean corpuscular hemoglobin concentration, MCHC; red cell distribution width–coefficient of variation, RDW-C; red cell distribution width–standard deviation, RDW-S; platelet count, PLT; mean platelet volume, MPV; platelet hematocrit, PCT; platelet distribution width, PDW; platelet large cell ratio, PLCR) were performed immediately after capillary blood sampling on the hematology analyzer Mythic® 18 (Orphée, Switzerland). Additionally at each TP analyses of biochemical indices (concentration of glucose, Glu; calcium, Ca; magnesium, Mg, phosphates, P) were performed from serum following capillary blood samples immediately after centrifugation (4000 × *g* for 10 min at 4 °C) on the ACCENT 220S automatic biochemical analyzer (Cormay, Łomianki, Poland). Moreover, each pre-supplementation blood sample was used for the determination of the activity of creatine kinase (CK), alanine aminotransferase (AlAT), aspartate aminotransferase (AspAT), lactate dehydrogenase (LDH), and concentration of urea (UREA) and creatinine (CREA), which served as the study characteristic data (analyzed by the same method as biochemical indices) (Table 2). Moreover, cellular components (WBC, LYM, MON, GRA, RBC, PLT), HGB and biochemical indices were calculated by taking into account the HCT conversion factor, following the methodology previously established in our studies [18,19,23–25]. This approach was applied to avoid misinterpretation of blood indices' results due to the HCT shifts. The correction was performed using a hypothetical hematocrit value of 0.500 L/L (50%), according to the methodology previously described by Podgórski et al. [24], to facilitate standardized comparisons of hematological variables between samples.

**Table 2.** Statistical results of GLM and Bonferroni post hoc analysis of hematological indices.

Indicator	‘TR’ × ‘TP’	‘TR’	‘TP’	Post hoc
WBC [10^9^/L]	F = 1.43 *p*-value = 0.224 η_*p*_^2^ = 0.05	F = 0.50 *p*-value = 0.665 η*_p_* ^2^ = 0.02	F = 280.38 *p*-value < 0.001 η_*p*_^2^ = 0.92	‘TR’ × ‘TP’: - ‘TR’: - ‘TP’: 0′ < 30′; 0′ < 60′; 0′ < 90′; 30′ < 60′; 30′ < 90′; 60′ < 90′;
LYM [10^9^/L^1^]	F = 1.57 *p*-value = 0.149 η_*p*_^2^ = 0.06	F = 2.32 *p*-value = 0.081 η_*p*_^2^ = 0.08	F = 328.29 *p*-value < 0.001 η_p_^2^ = 0.93	‘TR’ × ‘TP’: - ‘TR’: - ‘TP’: 0′ < 30′; 0′ < 60′; 0′ < 90′; 30′ < 60′; 30′ < 90′; 60′ < 90′;
MON [10^9^/L]	F = 1.04 *p*-value = 0.397 η_*p*_^2^ = 0.04	F = 0.95 *p*-value = 0.440 η_*p*_^2^ = 0.04	F = 137.82 *p*-value < 0.001 η_*p*_^2^ = 0.85	‘TR’ × ‘TP’: - ‘TR’: - ‘TP’: 0′ < 30′; 0′ < 60′; 0′ < 90′; 30′ < 60′; 30′ < 90′; 60′ < 90′;
GRA [10^9^/L]	F = 1.53 *p*-value = 0.212 η_*p*_^2^ = 0.06	F = 0.30 *p*-value = 0.874 η_*p*_^2^ = 0.01	F = 106.20 *p*-value < 0.001 η_*p*_^2^ = 0.81	‘TR’ × ‘TP’: - ‘TR’: - ‘TP’: 0′ < 60′; 0′ < 90′; 30′ < 90′; 60′ < 90′;
LYM [%]	F = 0.87 *p*-value = 0.524 η_*p*_^2^ = 0.03	F = 2.22 *p*-value = 0.072 η_*p*_^2^ = 0.08	F = 181.41 *p*-value < 0.001 η_*p*_^2^ = 0.88	‘TR’ × ‘TP’: - ‘TR’: - ‘TP’: 0′ < 30′; 0′ < 60′; 0′ < 90′; 30′ < 90′; 60′ < 90′;
MON [%]	F = 1.54 *p*-value = 0.166 η_*p*_^2^ = 0.06	F = 0.90 *p*-value = 0.432 η_*p*_^2^ = 0.03	F = 2.34 *p*-value = 0.126 η_p_^2^ = 0.09	‘TR’ × ‘TP’: - ‘TR’: - ‘TP’: -
GRA [%]	F = 0.89 *p*-value = 0.506 η_*p*_^2^ = 0.03	F = 1.83 *p*-value = 0.129 η_*p*_^2^ = 0.07	F = 177.99 *p*-value < 0.001 η_*p*_^2^ = 0.88	‘TR’ × ‘TP’: - ‘TR’: - ‘TP’: 30′ < 0′; 60′ < 0′; 90′ < 0′; 90′ < 30′; 90′ < 60′;
RBC [10^12^/L]	F = 0.53 *p*-value = 0.672 η_*p*_^2^ = 0.02	F = 0.62 *p*-value = 0.569 η_*p*_^2^ = 0.03	F = 52.18 *p*-value < 0.001 η_*p*_^2^ = 0.68	‘TR’ × ‘TP’: - ‘TR’: - ‘TP’: 90′ < 0′; 90′ < 30′; 90′ < 60′;
HGB [mmol/L]	F = 0.33 *p*-value = 0.922 η_*p*_^2^ = 0.01	F = 6.57 *p*-value < 0.001 η_*p*_^2^ = 0.21	F = 58.20 *p*-value < 0.001 η_*p*_^2^ = 0.70	‘TR’ × ‘TP’: - ‘TR’: *BASE < LOW; BASE < MEDIUM; BASE < HIGH; BASE < PLA*; ‘TP’: 30′ < 0′; 60′ < 0′; 90′ < 0′; 90′ < 30′; 90′ < 60′;
HCT [L/L]	F = 1.96 *p*-value = 0.068 η_*p*_^2^ = 0.07	F = 2.71 *p*-value = 0.035 η_*p*_^2^ = 0.10	F = 37.55 *p*-value < 0.001 η_*p*_^2^ = 0.60	‘TR’ × ‘TP’: - ‘TR’: *PLA < BASE*; ‘TP’: 0′ < 90′; 30′ < 90′; 60′ < 90′;
MCV [fL]	F = 0.57 *p*-value = 0.620 η_*p*_^2^ = 0.02	F = 0.75 *p*-value = 0.506 η_*p*_^2^ = 0.03	F = 71.45 *p*-value < 0.001 η_*p*_^2^ = 0.74	‘TR’ × ‘TP’: - ‘TR’: - ‘TP’: 0′ < 90′; 30′ < 90′; 60′ < 90′;
MCH [fmol]	F = 0.39 *p*-value = 0.883 η_*p*_^2^ = 0.02	F = 3.21 *p*-value = 0.031 η_*p*_^2^ = 0.11	F = 35.28 *p*-value < 0.001 η_*p*_^2^ = 0.59	‘TR’ × ‘TP’: - ‘TR’: *BASE < LOW; BASE < PLA*; ‘TP’: 30′ < 0′; 60′ < 0′; 90′ < 0′ 90′ < 30′;
MCHC [mmol/L]	F = 0.40 *p*-value = 0.884 η_*p*_^2^ = 0.02	F = 6.54 *p*-value < 0.001 η_*p*_^2^ = 0.21	F = 57.83 *p*-value < 0.001 η_*p*_^2^ = 0.70	‘TR’ × ‘TP’: - ‘TR’: *BASE < LOW; BASE < MEDIUM; BASE < HIGH; BASE < PLA;* ‘TP’: 30′ < 0′; 60′ < 0′; 90′ < 0′; 90′ < 30′; 90′ < 60′;
RDW-C [%]	F = 1.00 *p*-value = 0.447 η_*p*_^2^ = 0.04	F = 0.40 *p*-value = 0.808 η_*p*_^2^ = 0.02	F = 4.17 *p*-value = 0.019 η_*p*_^2^ = 0.14	‘TR’ × ‘TP’: - ‘TR’: - ‘TP’: 0′ < 60′;
RDW-S [fL]	F = 1.75 p-value = 0.132 η_p_^2^ = 0.07	F = 1.53 *p*-value = 0.198 η_*p*_^2^ = 0.06	F = 16.46 *p*-value < 0.001 η_*p*_^2^ = 0.40	‘TR’ × ‘TP’: - ‘TR’: - ‘TP’: 0′< 90′; 30′ < 90′; 60′ < 90′;
PLT [10^9^/L]	F = 1.53 *p*-value = 0.112 η_*p*_^2^ = 0.06	F = 2.47 *p*-value = 0.049 η_p_^2^ = 0.09	F = 34.53 *p*-value < 0.001 η_*p*_^2^ = 0.58	‘TR’ × ‘TP’: - ‘TR’: - ‘TP’: 30′ < 0′; 60′< 0′; 90′ < 0′; 90′ < 60′;
MPV [fL]	F = 1.05 *p*-value = 0.405 η_*p*_^2^ = 0.04	F = 0.26 p-value = 0.852 η_*p*_^2^ = 0.01	F = 24.91 *p*-value < 0.001 η_*p*_^2^ = 0.50	‘TR’ × ‘TP’: - ‘TR’: - ‘TP’: 0′ < 90′; 30′ <90′; 60′<90′;
PCT [cl/L]	F = 0.70 *p*-value = 0.753 η_*p*_^2^ = 0.03	F = 0.48 *p*-value = 0.753 η_*p*_^2^ = 0.03	F = 22.39 *p*-value < 0.001 η_*p*_^2^ = 0.47	‘TR’ × ‘TP’: - ‘TR’: - ‘TP’: 30′<0′; 60′<0′; 90′<0′;
PDW [%]	F = 0.45 *p*-value = 0.875 η_*p*_^2^ = 0.02	F = 1.40 *p*-value = 0.239 η_*p*_^2^ = 0.05	F = 9.05 *p*-value < 0.001 η*_p_* ^2^ = 0.27	‘TR’ × ‘TP’: - ‘TR’: - ‘TP’: 30′ < 0′; 60′ < 0′; 90′ < 0′;
PLCR [%]	F = 1.84 *p*-value = 0.087 η_*p*_^2^ = 0.07	F = 0.85 *p*-value = 0.499 η_*p*_^2^ = 0.03	F = 36.84 *p*-value < 0.001 η_*p*_^2^ = 0.60	‘TR’ × ‘TP’: - ‘TR’: - ‘TP’: 0′ < 30′; 0′ < 60′; 0′ < 90′; 30′ < 90′; 60′ < 90′;

WBC–white blood cells; LYM–lymphocytes; MON–monocytes; GRA–granulocytes; RBC–red blood cells count; HGB–hemoglobin concentration; HCT–hematocrit; MCV–mean corpuscular volume; MCH–mean corpuscular hemoglobin mass; MCHC–mean corpuscular hemoglobin concentration; RDW-C–red cell distribution width–coefficient of variation; RDW-S–red cell distribution width–standard deviation; MPV–mean platelet volume; PCT–platelet hematocrit; PDW–platelet distribution width; PLT–platelet count; PLCR–platelet large cell ratio; ‘TR’ × ‘TP’–interaction effect of treatment and time point; ‘TR’–main effect of treatment; ‘TP’–main effect of time point; Treatment conditions: *BASE*, baseline; *LOW*, 3 mg_CAF_/kg_BM_; *MEDIUM*, 6 mg_CAF_/kg_BM_; *HIGH*, 9 mg_CAF_/kg_BM_; *PLA*, placebo. Time points: 0′, pre-supplementation; 30′, 30 min after supplementation; 60′, pre-exercise; 90′, 3 min post-exercise; *F*–test statistic; *η*_*p*_^2^–partial eta squared.

#### 2.3.5. Statistical analysis

The analysis was performed using Statistica 13.3 (TIBCO Software Inc., 2017). Data were inspected for potential outliers and missing values. Missing values, which accounted for less than 0.15% of the total dataset, were imputed using the k-nearest neighbors algorithm to preserve the underlying data structure and temporal dependencies. Potential outliers were identified separately for each biochemical variable using the 1.5 × interquartile range (IQR) criterion. Overall, 80 outlying observations (0.64% of 12.480 observations) were flagged as potential outliers across 23 variables; their distribution is provided in Supplementary materials, Table S2. Each flagged observation was subsequently inspected individually to distinguish potential measurement or data-entry errors from physiologically plausible extreme responses. Following this review, observations considered unreliable were treated as missing values and handled using the same k-nearest neighbors imputation procedure. In one case, a participant exhibiting an exceptionally high number of outlying Glu measurements was excluded from Glu-specific analyses because the Glu profile was considered unreliable; however, this participant remained included in analyses of all other biochemical variables. This procedure was adopted to minimize the influence of extreme observations while preserving the maximum amount of valid data.

Blood indices were analyzed using a repeated-measures analysis of variance within the GLM framework, to detect potential differences influenced by: (a) the ingestion of different doses of CAF and *PLA* or *BASE* condition (main factor: *treatment* [‘TR’]); (b) changes over time during a single study visit, with an additional intervention in the form of the CrossFit® workout (main factor: *time point* [‘TP’]); and (c) the interaction between the two factors (‘TR’ × ‘TP’). The normality of the variable distributions was tested by comparing the values of skewness and kurtosis with their standard errors [25]. The sphericity of the variables was assessed using Mauchly's test, and if violated, the Greenhouse–Geisser correction was applied. Post hoc analyses were performed using the Bonferroni test to identify specific group differences where main effects or interactions were significant. The data are presented as means with standard deviation (*SD*). The level of significance for all statistical analyses was set at *α* = 0.05. Effect sizes were reported as partial eta squared (*η*_*p*_^2^), with ranges for interpretation as follows: 0.01–0.06 (small effect), 0.06–0.14 (moderate effect), and >0.14 (large effect). Sample size estimation was performed using G*Power software (version 3.1.9.6, Germany). The sample size calculation was conducted for the primary outcome of the original randomized crossover trial, namely the CrossFit® exercise performance, which has been reported previously [16]. Based on the umbrella review by Grgic et al. [26], a moderate effect size (f(U) = 0.38) was assumed for a repeated-measures ANOVA (within-subject factors). The calculation assumed one group, five repeated measurements, *α* = 0.05, statistical power > 0.80, and a nonsphericity correction (*ε*) of 1.0. Under these assumptions, the required sample size was 23 participants. To account for potential attrition, 26 participants were recruited and completed the study.

## 3. Results

The absence of statistically significant differences for all analyzed variables at the first –0′ TP (pre-supplementation) of each study visit (T_0_–T_4_) (corresponding to the treatment condition) indicates that the baseline concentrations of the studied variables were comparable across all study visits for each participant (Supplementary materials, Table S1). This finding supports the adequacy of the adopted wash-out period and justifies proceeding with further analyses under the assumption that any potential carry-over effects have been minimized.

### 3.1. Hematological results

GLM analysis showed no statistical significance for the ‘TR’ × ’TP’ interaction effect of any hematological variable. Significance for the main effect ‘TR’ was observed only for HGB, HCT, MCH, MCHC, and PLT variables. Main effect ‘TP’ showed significant differences for all hematological variables except MON%. All data of GLM analysis results, and Bonferroni post hoc analysis results are presented in the Table 2. Due to the large volume of data, the descriptive statistics (Means ± SD) for all TP and conditions are provided in Supplementary materials (Table S1).

WBC, LYM, and MON were characterized by an increase in counts over time (a significant difference with each subsequent TP). A similar pattern was observed for GRA, LYM%, and PLCR, although no significant differences were found between 30′ and 60′ TP. No dose-related differences were observed, and MON% remained unchanged across TP.

For GRA%, HGB, and MCHC, a decrease in values and concentrations was observed with subsequent TP on the test day. A similar phenomenon was observed for MCH and PLT (no significant difference between 30′ and 90′ TP).

For HCT, MCV, RDW-S, and MPV, 90′ TP (post-exercise) revealed significantly higher concentrations compared to other TP. However, RBC count was significantly lower at 90′ TP than at the TPs.

For RDW-C, the only difference was observed between 0′ TP and 60′ TP (*p*-value = 0.007). In turn, for PCT and PDW, the highest values were observed at 0′ TP, compared to all other TP.

Bonferroni post hoc for the significant ‘TR’ factor indicated that for HGB, significantly lower values were observed for *BASE* compared to the results after *LOW* (*p*-value = 0.001), *MEDIUM* (*p*-value < 0.001), and *HIGH* (*p*-value = 0.004) CAF dose, or *PLA* (*p*-value = 0.001) intake. For HCT, the only difference was observed in significantly lower concentrations of this variable for *PLA* compared to *BASE* (*p*-value = 0.021). MCH was significantly lower at *BASE* compared to *LOW* (*p*-value = 0.032) and *PLA* (*p*-value = 0.040). MCHC was also significantly lower at *BASE* compared to *LOW* (*p*-value = 0.001), *MEDIUM* (*p*-value < 0.001), and *HIGH* (*p*-value = 0.004), as well as *PLA* (*p*-value = 0.001).

Despite the observed significance of the main factor ‘TR’ for PLT, post hoc analysis indicated no significant differences.

### 3.2. Biochemical results

GLM analysis showed statistical significance for the ‘TR’ × ‘TP’ interaction effect only for Glu concentration. Significance for the main effect ‘TR’ was observed only for P concentration. Main effect ‘TP’ showed significant changes for Ca and P concentrations. All data of means with SD, GLM analysis results, and Bonferroni post hoc analysis results are presented in the Table 3.

**Table 3.** Means with standard deviations (SD) and statistical results of GLM and Bonferroni post hoc analysis of biochemical variables.

*Glucose* [mg/dL]									
**Treatment**	**1**	**2**	**3**	**4**	**‘TR’ mean**	**‘TR’ × ‘TP’**	**‘TR’**	**‘TP’**	**Post hoc**
*BASE*	123.27 (17.44)	125.47 (19.09)	117.58 (15.21)	180.59 (51.18)	136.73	*F* = 3.25 *p-*value = 0.006 *η*_*p*_^*2*^ = 0.12	*F* = 4.23 *p-*value = 0.003 *η*_*p*_^*2*^ = 0.15	*F* = 55.08 *p-*value < 0.001 *η*_*p*_^*2*^ = 0.70	‘TR’ × ‘TP’: *BASE_*0′ < *BASE_*90′; *BASE_*30′ < *BASE_*90′; *BASE_*60′ < *BASE_*90′; *BASE_*90′ < *MEDIUM_*90′; *BASE_*90′ < *HIGH_*90′; *LOW_*0′ < *LOW*_90′; *LOW_*30′ < *LOW_*90′; *LOW_6*0′ < *LOW_*90′; *MEDIUM_*0′ < *MEDIUM_*90′; *MEDIUM_*30′ < *MEDIUM_*90′; *MEDIUM_*60′ < *MEDIUM_*90′; *HIGH_*0′ < *HIGH_*90′; *HIGH_*30′ < *HIGH_*90′; *HIGH_*60′ < *HIGH_*90′; *PLA_*0′ < *PLA_*90′; *PLA_*30′ < *PLA_*90′; *PLA_*60′ < *PLA_*90′; *PLA_*90′ < *MEDIUM_*90′; *PLA_*90′ < *HIGH_*90′; ‘TR’: *BASE < MEDIUM; BASE < HIGH*; ‘TP’: 0′ < 90′; 30′ < 90′; 60′ < 90′;
*LOW*	127.99 (21.65)	132.03 (21.36)	118.58 (12.38)	197.03 (54.87)	143.57				
*MEDIUM*	121.78 (19.54)	134.38 (19.91)	119.78 (15.60)	209.31 (52.71)	145.43				
*HIGH*	129.42 (20.33)	134.39 (20.64)	121.03 (15.12)	201.97 (51.18)	145.82				
*PLA*	127.65 (23.47)	134.58 (20.27)	120.82 (13.92)	180.90 (47.44)	140.99				
‘TP’ mean	126.02	132.17	119.56	192.28					
*Calcium* [mg/dL]									
**Treatment**	**1**	**2**	**3**	**4**	**‘TR’ mean**	**‘TR’ × ‘TP’**	**‘TR’**	**’TP’**	**Post hoc**
*BASE*	11.76 (1.12)	12.10 (1.08)	11.88 (1.05)	11.75 (1.00)	11.88	*F* = 0.64 *p-*value = 0.810 *η*_*p*_^*2*^ = 0.02	*F* = 0.50 *p-*value = 0.739 *η*_*p*_^*2*^ = 0.02	*F* = 11.91 *p-*value < 0.001 *η*_*p*_^*2*^ = 0.32	‘TR’ × ‘TP’: - ‘TR’: - ‘TP’: 0′ < 30′; 90′ < 30′; 90′ < 60′;
*LOW*	11.94 (1.03)	12.03 (0.97)	11.94 (0.95)	11.84 (0.91)	11.93				
*MEDIUM*	11.93 (0.85)	12.16 (0.94)	12.03 (0.98)	11.85 (1.13)	11.91				
*HIGH*	11.84 (0.93)	12.12 (1.19)	12.08 (0.96)	11.65 (0.98)	11.92				
*PLA*	11.93 (0.84)	12.21 (1.04)	12.12 (0.82)	11.77 (0.88)	12.01				
‘TP’ mean	11.88	12.12	12.01	11.77					
*Magnesium* [mmol/L]									
**Treatment**	**1**	**2**	**3**	**4**	**‘TR’ mean**	**‘TR’ × ‘TP’**	**‘TR’**	**’TP’**	**Post hoc**
*BASE*	1.00 (0.11)	1.01 (0.12)	0.98 (0.13)	1.00 (0.10)	0.99	*F* = 1.49 *p-*value = 0.127 *η*_*p*_^*2*^ = 0.06	*F* = 1.30 *p-*value = 0.277 *η*_*p*_^*2*^ = 0.05	*F* = 1.11 *p-*value = 0.351 *η*_*p*_^*2*^ = 0.04	‘TR’ × ‘TP’: - ‘TR’: - ‘TP’: -
*LOW*	0.99 (0.12)	0.99 (0.11)	1.01 (0.16)	1.00 (0.12)	1.00				
*MEDIUM*	1.00 (0.12)	1.00 (0.13)	1.00 (0.12)	1.00 (0.13)	1.00				
*HIGH*	0.98 (0.11)	0.99 (0.12)	0.97 (0.11)	0.97 (0.11)	0.98				
*PLA*	1.00 (0.13)	1.02 (0.14)	1.01 (0.13)	0.98 (0.12)	1.01				
‘TP’ mean	0.99	1.00	0.99	0.99					
*Phosphates* [mg/dL]									
**Treatment**	**1**	**2**	**3**	**4**	**‘TR’ mean**	**‘TR’ × ‘TP’**	**‘TR’**	**‘TP’**	**Post hoc**
*BASE*	4.51 (0.78)	4.75 (0.86)	4.81 (1.28)	6.65 (1.05)	5.24	*F* = 1.44 *p-*value = 0.146 *η*_*p*_^2^ = 0.05	*F* = 2.95 *p-*value = 0.024 *η*_*p*_^2^ = 0.11	*F* = 179.15 *p-*value < 0.001 *η*_*p*_^2^ = 0.88	‘TR’ × ‘TP’: - ‘TR’: *BASE < MEDIUM*; ‘TP’: 0′ < 60′; 0′ < 90′; 30′ < 90′; 60′ < 90′;
*LOW*	4.75 (0.91)	4.85 (1.03)	5.30 (1.32)	6.88 (1.24)	5.44				
*MEDIUM*	4.98 (0.68)	5.12 (0.86)	5.13 (0.89)	7.43 (1.33)	5.62				
*HIGH*	4.69 (0.96)	4.78 (1.02)	4.90 (0.95)	6.83 (1.19)	5.30				
*PLA*	4.68 (0.71)	4.95 (0.93)	5.33 (1.05)	6.89 (1.04)	5.48				
‘TP’ mean	4.74	4.89	5.13	6.90					

‘TR’ × ‘TP’–interaction effect of treatment and time point; ‘TR’–main effect of treatment; ‘TP’–main effect of time point; *BASE*–baseline (no supplementation); *LOW*–3 mg_CAF_/kg_BM_; *MEDIUM*–6 mg_CAF_/kg_BM_; *HIGH*–9 mg_CAF_/kg_BM_; *PLA*–placebo; 0′–pre-supplementation; 30′–30 min after supplementation; 60′–pre-exercise; 90′–3 min post-exercise; ‘TP’ mean–marginal means for time points; ‘TR’ mean–marginal means for treatments; *F*–test statistic; η_*p*_^2^–partial eta squared.

Glu concentration was highest at the final (90′) TP during all study visits (*p*-value < 0.001). In addition, significantly higher values were observed after supplementation with *MEDIUM* (*p*-value = 0.011) and *HIGH* (*p*-value = 0.007) doses of CAF, compared to *BASE*. A detailed post hoc Bonferroni analysis of the ‘TR’ × ‘TP’ interaction revealed that significant differences were present only for 90′ TP, between *MEDIUM* and *HIGH* doses of CAF, compared to *BASE* and *PLA* (*MEDIUM* > *BASE*: *p*-value < 0.001; *MEDIUM* > *PLA*: *p*-value < 0.001; *HIGH* > *BASE*: *p*-value = 0.014; *HIGH* > *PLA*: *p*-value = 0.019). The highest Glu concentrations at 90′ TP occurred following the *MEDIUM* dose of CAF; however, these values did not differ significantly from those observed in *LOW* and *HIGH* conditions.

Ca concentration was characterized by the highest value at 30′ TP after CAF consumption. At this TP, Ca concentrations were significantly higher than at 0′ TP (*p*-value = 0.001) and 90′ TP (*p*-value < 0.001), but there was no significant difference between Ca concentrations at 30′ and 60′ TP. In addition, Ca at 60′ TP was significantly higher than at the 90′ TP (*p*-value = 0.002), immediately after exercise, but was not significantly different from the 0′ TP, although its concentration was higher.

For Mg, no significant differences were observed under the influence of supplement intake, ‘TP’, or in the ‘TR’ × ‘TP’ interaction.

As for P, its highest concentration was observed at *MEDIUM*. It was significantly higher compared to *BASE* (*p*-value = 0.027). No differences were observed between other doses and *PLA*. In addition, post hoc analysis for the main factor ‘TP’ showed that P concentrations observed immediately after the CrossFit® workout (90′ TP) were significantly higher compared to BASE at 0′, 30′, and 60′ TP (*p*-value < 0.001 for each comparison). Moreover, P at 60′ TP was significantly higher than at 0′ TP (*p*-value = 0.002).

### 3.3. Sensitivity analysis

Overall, the sensitivity analysis showed a high degree of consistency with the primary analysis. Most ‘TR’ × ‘TP’, ‘TR’, and ‘TP’ effects retained their original significance status, with the complete comparison of omnibus effects presented in Supplementary materials Table S3 and the effects showing changes in statistical significance summarized in Table 4.

**Table 4.** Effects showing a change in statistical significance between the primary and sensitivity analyses excluding the *BASE* condition.

Variable – effect	Primary analysis	Sensitivity analysis (*BASE* excluded)	Δη_*p*_^2^	Interpretation
HGB–‘TR’	*F* = 6.57, *p*-value < 0.001 *η*_*p*_^2^ = 0.208	*F* = 0.08 *p*-value = 0.968 *η*_*p*_^2^ = 0.003	−0.205	‘TR’ effect disappears
HCT–‘TR’	*F* = 2.71 *p*-value = 0.035 *η*_*p*_^2^ = 0.098	*F* = 1.74 *p*-value = 0.166 *η*_*p*_^2^ = 0.065	−0.033	‘TR’ effect disappears
MCH–‘TR’	*F* = 3.21 *p*-value = 0.031 *η*_*p*_^2^ = 0.114	*F* = 0.17 *p*-value = 0.918 *η*_*p*_^2^ = 0.007	−0.107	‘TR’ effect disappears
MCHC–‘TR’	*F* = 6.54 *p*-value < 0.001 *η*_*p*_^2^ = 0.207	*F* = 0.07 *p*-value = 0.977 *η*_*p*_^2^ = 0.003	−0.205	‘TR’ effect disappears
LYM%–‘TR’	*F* = 2.22 *p*-value = 0.072 *η*_*p*_^2^ = 0.082	*F* = 2.75 *p*-value = 0.048 *η*_*p*_^2^ = 0.099	+0.018	‘TR’ effect emerges
RDW-C–‘TP’	*F* = 4.17 *p*-value = 0.019 *η*_*p*_^2^ = 0.143	*F* = 2.15 *p*-value = 0.101 *η*_*p*_^2^ = 0.079	−0.064	‘TP’ effect disappears
Glucose–‘TR’	*F* = 4.23, *p*-value = 0.003 *η*_*p*_^2^ = 0.150	*F* = 1.47 *p*-value = 0.231 *η*_*p*_^2^ = 0.058	−0.092	‘TR’ effect disappears
Phosphates–‘TR’	*F* = 2.95 *p*-value = 0.024 *η*_*p*_^2^ = 0.110	*F* = 2.34 *p*-value = 0.080 *η*_*p*_^2^ = 0.086	−0.024	‘TR’ effect disappears

LYM–lymphocytes; HGB–hemoglobin concentration; HCT–hematocrit; MCH–mean corpuscular hemoglobin mass; MCHC–mean corpuscular hemoglobin concentration; RDW-C–red cell distribution width–coefficient of variation; ‘TR’–main effect of treatment; ‘TP’–main effect of time point; Treatment conditions: BASE, baseline; F–test statistic; η_p_^2^–partial eta squared; Δη*_p_* ^2^–difference in partial eta squared between the sensitivity and primary analyses.

The sensitivity analysis excluding the *BASE* condition largely confirmed the primary findings for time-dependent changes. The ‘TR’ × ‘TP’ interaction remained non-significant across the hematological indices, while the main effect of ‘TP’ remained significant for most hematological indices. In contrast, the previously observed ‘TR’ effects for HGB, HCT, MCH, and MCHC were no longer significant after excluding *BASE*, suggesting that these effects were primarily attributable to differences between the *BASE* condition and the experimental visits rather than to differences among CAF doses and *PLA*. A ‘TR’ effect emerged for LYM%, with Bonferroni-adjusted post hoc analysis indicating higher LYM% in the *HIGH* condition compared with *PLA* (*p =* 0.045). Given the borderline omnibus and pairwise *p*-values and the absence of a corresponding treatment effect in the primary analysis, this isolated finding should be interpreted cautiously. Additionally, the previously significant main effect of ‘TP’ for RDW-C was no longer significant after excluding BASE (*p* = 0.101).

For biochemical variables, the sensitivity analysis largely confirmed the primary findings for Glu and P. For Glu, the ‘TR’ × ‘TP’ interaction remained significant, as did the main effect of ‘TP’, whereas the previously observed main effect of ‘TR’ was no longer significant. Importantly, Bonferroni-adjusted post hoc comparisons of the ‘TR’ × ‘TP’ interaction confirmed that post-exercise Glu concentrations remained significantly higher in the *MEDIUM* and *HIGH* conditions than in *PLA*, while no significant differences were observed among the CAF doses. Thus, although the overall main effect of ‘TR’ appears to have been partly attributable to the inclusion of the *BASE* visit, the differential temporal response for Glu across the experimental conditions—and specifically the greater post-exercise Glu concentrations in the *MEDIUM* and *HIGH* conditions compared with *PLA*—remained evident after *BASE* exclusion. For P, the ‘TR’ × ‘TP’ interaction remained non-significant and the strong main effect of ‘TP’ was preserved, whereas the previously significant main effect of ‘TR’ was no longer observed after excluding *BASE*. Thus, the ‘TR’ effect for P also appears to have been largely attributable to differences between the *BASE* condition and the experimental visits rather than differences among CAF doses and *PLA*.

## 4. Discussion

The present study examined the effects of acute CAF ingestion at three different doses (3, 6, and 9 mg _CAF_/kg_BM_) and *PLA* on hematological and biochemical indices during the standardized CrossFit®-specific workout protocol, FGB. The principal findings indicate that: (1) exercise was the predominant driver of hematological changes, with no CAF-specific modulation of blood cell indices; (2) significant ‘TR’ × ‘TP’ interactions were observed solely for Glu concentration, with *MEDIUM* and *HIGH* CAF doses producing significantly greater post-exercise Glu elevations compared to *BASE* and *PLA*; (3) P concentration was specifically elevated with *MEDIUM* CAF dose across all TP and increased markedly post-exercise; (4) Ca concentration increased following supplementation (regardless of content, from the first to the second—30′ TP) and declined post-exercise, with no CAF-specific effects; and (5) Mg remained stable across all conditions. The observed differences in HGB, HCT, MCH, and MCHC between *BASE* visit and subsequent experimental visits (including *PLA*) likely reflect methodological factors such as familiarization rather than pharmacological effects of CAF. Overall, these findings demonstrate that the acute physiological effects of CAF during CrossFit® are highly selective, primarily manifesting as alterations in post-exercise Glu responses, with minimal impact on hematological homeostasis.

### 4.1. Absence of CAF-specific hematological effects

No significant ‘TR’ × ‘TP’ interactions were identified for any hematological variable, indicating that CAF supplementation did not differentially modulate the time course of exercise-induced changes in these blood indices. While significant main effects of ‘TR’ were detected for HGB, HCT, MCH, MCHC, and PLT, post hoc analyses revealed that these differences primarily reflected lower values during *BASE* visit compared to subsequent experimental sessions—including *PLA*. The fact that HGB, MCH, and MCHC were lower at *BASE* compared to all subsequent conditions strongly suggests that these differences reflect visit-specific factors rather than pharmacological effects of CAF. These findings extend current knowledge by demonstrating that even when significant ‘TR’ main effects are detected, careful examination of post hoc patterns and inclusion of *PLA* controls are essential to distinguish true pharmacological effects from methodological artifacts.

### 4.2. RBC and PLT responses to exercise

Exercise induced complex, time-dependent changes in RBC and PLT indices that were independent of CAF supplementation. Post-exercise (90′) TP was characterized by significantly higher HCT, MCV, RDW-S, and MPV compared to all other TP, while RBC count was significantly lower post-exercise. These findings suggest exercise-induced hemoconcentration, associated with acute plasma volume shifts, and are consistent with previous reports [27,28]. The post-exercise decrease in RBC count coupled with increases in HCT and MCV appears paradoxical but may reflect several underlying mechanisms. First, high-intensity exercise can induce transient splenic contraction, releasing a population of larger, younger RBCs into circulation, thereby increasing MCV without proportionally increasing RBC count [29]. Second, mechanical hemolysis—particularly during high-impact, repetitive movements characteristic of CrossFit® – may selectively disrupt older, more fragile RBCs, leaving a population with higher mean volume [30]. Third, fluid shifts and plasma volume contraction during intense exercise can increase HCT while simultaneously concentrating metabolic byproducts that influence RBC morphology [12,31]. Kashef et al. [9]. reported contrasting findings in professional male CrossFit® athletes, observing decreases in RBC, HCT, HGB, MCV, MCH, and MCHC following a single CrossFit® session, alongside increases in WBC and PLT. These discrepancies likely reflect differences in sampling methodology (venous vs. capillary blood), plasma volume correction approaches (the present study applied HCT conversion factors to cellular components), participant training status, and specific workout protocols [9,12]. The observed post-exercise HCT elevation is more consistent with classical exercise physiology documenting hemoconcentration during high-intensity efforts, particularly when plasma volume correction is not applied [32,33]. The observed increases in MPV and PDW post-exercise are consistent with exercise-induced thrombocytosis and PLT activation. The increase in MPV reflects the release of larger, more metabolically active PLT from bone marrow and spleen in response to exercise-induced catecholamine surges and shear stress [34,35]. PLT activation during high-intensity exercise serves adaptive hemostatic functions but may also contribute to acute thrombotic risk in susceptible individuals [36]. The progressive increase in PLCR throughout the testing session further supports ongoing PLT activation. Importantly, CAF supplementation did not modulate these PLT responses, contrasting with *in vitro* evidence suggesting that CAF may influence PLT aggregation via adenosine receptor mechanisms [37].

### 4.3. Exercise-induced leukocytosis and immune cell mobilization

In contrast to the absence of CAF effects, exercise exerted profound and consistent effects on leukocyte dynamics. WBC, LYM, MON, GRA counts increased progressively across TP, with significant elevations at each successive measurement. This pattern reflects the well-characterized phenomenon of exercise-induced leukocytosis, driven by the rapid mobilization of immune cells from marginated pools, lymphoid organs, and bone marrow in response to catecholamine surges, shear stress, and inflammatory signaling [10,38]. Adammek et al. [10] provided detailed kinetic data demonstrating that circulating leucocytes, LYM and neutrophils increase within 5 min of moderate-to-vigorous aerobic exercise, with continued mobilization throughout the exercise bout. The magnitude of these responses is strongly influenced by exercise intensity and duration, with high-intensity intermittent protocols—such as the CrossFit® workout implemented in our study—eliciting particularly pronounced elevations [39,40]. The observed increase in absolute GRA counts alongside a decrease in GRA percentage reflects the proportionally greater mobilization of LYM and MON relative to GRA during the initial phase of the exercise response, consistent with the biphasic model of exercise immunology [38,40]. Critically, CAF supplementation did not modulate the magnitude or time course of exercise-induced leukocytosis, as evidenced by the absence of ‘TR’ × ‘TP’ interactions for WBC and differential counts. This contrasts with emerging evidence suggesting that CAF may exert genotype-dependent immunomodulatory effects. Rahimi and Ahmetov [15] reported that individuals with the *ADORA2A* TT genotype exhibited reduced inflammatory markers (myeloperoxidase and acetylcholinesterase) following resistance exercise with 6 mg/kg_BM_ CAF supplementation, indicating that adenosine receptor polymorphisms may mediate the anti-inflammatory actions of CAF.

### 4.4. Glu metabolism following acute CAF supplementation

Glu concentration was the only variable exhibiting a significant ‘TR’ × ‘TP’ interaction among all hematological and biochemical indices analyzed. Post hoc analysis revealed that the interaction was driven entirely by post-exercise (90′ TP) differences, with *MEDIUM* and *HIGH* CAF doses eliciting significantly greater Glu concentrations compared with both *BASE* and *PLA* conditions. Although the *LOW* dose did not significantly differ from *BASE* and *PLA*, direct statistical comparisons between the three CAF doses were not significant. Therefore, these findings indicate that significant post-exercise Glu elevations were observed following the 6 and 9 mg_CAF_/kg_BM_ conditions compared with BASE and PLA, but they do not provide evidence for a dose-dependent, threshold, or ceiling effect. These findings align closely with previous CrossFit® research, where Fogaça et al. [7] reported that acute CAF supplementation (6 mg _CAF_/kg_BM_) increased post-workout Glu by approximately 3.2 mmol/L compared to *PLA* in trained CrossFit® males. These observations are further extended by demonstrating that the Glu response to CAF was confined to the post-exercise period, whereas no significant differences were observed during the pre-exercise measurements or between the CAF doses. The mechanisms by which CAF increases post-exercise Glu concentrations are multifactorial and may include enhanced hepatic Glu output [41], catecholamine-mediated metabolic stimulation [42], reduced insulin sensitivity and altered Glu uptake [43] or shifted substrate utilization during exercise [4]. Graham et al [4]. provided a comprehensive mechanistic review demonstrating that CAF (5–9 mg_CAF_/kg_BM_) influences skeletal muscle carbohydrate and fat metabolism through multiple pathways, with net effects varying based on dose, timing, exercise intensity, and individual metabolic phenotype. These findings further extend the available literature by demonstrating that, within a CrossFit® context both the *MEDIUM* and *HIGH* CAF doses elicited significant post-exercise Glu elevations compared with *BASE* and *PLA*, whereas no significant differences were observed between the two CAF doses. Although the observed post-exercise Glu elevations demonstrate a measurable physiological response to CAF supplementation, this study was not designed to determine whether the magnitude of this response is sufficient to confer meaningful practical or performance-related benefits during a CrossFit® exercise. Because Glu kinetics, insulin concentrations, and tissue Glu uptake were not assessed, it is not possible to determine the relative contribution of enhanced hepatic Glu output, reduced Glu uptake, or altered substrate utilization to the observed Glu response. Therefore, higher post-exercise Glu concentrations should be interpreted as a pharmacological response to acute CAF ingestion rather than direct evidence of improved carbohydrate availability or enhanced exercise performance.

### 4.5. Ca: time-dependent changes without CAF modulation

Ca concentration exhibited a significant main effect of ‘TP’ but no ‘TR’ effect or ‘TR’ × ’TP’ interaction, indicating that temporal dynamics were independent of CAF supplementation. The highest Ca concentrations occurred at 30′ TP, significantly exceeding baseline and post-exercise values. Ca remained elevated at the pre-exercise TP compared to post-exercise but did not differ significantly from *BASE*. The observed increase in serum Ca following supplementation—regardless of whether the supplement contained CAF or *PLA*—likely reflects several mechanisms, like postprandial Ca absorption. The standardized pre-visit meal (consumed 3 h before testing) was individually adjusted to body mass (to provide 2 g of carbohydrates/kg_BM_ and 25 g of protein; it consistently consisted of skyr, strawberries, oat flakes, and banana, and contained around 160–200 mg of Ca) and the timing of supplementation (70 min pre-exercise) coincides with the expected peak of postprandial Ca absorption. The liquid supplement (orange juice and water) may have further facilitated Ca ionization and absorption [44,45]. Moreover, the decline in serum Ca immediately post-exercise aligns with established exercise physiology, because high-intensity exercise induces Ca sequestration into skeletal muscle sarcoplasmic reticulum to support excitation-contraction coupling; Ca buffering by plasma proteins due to acid-base shifts and altered protein binding; and potential Ca loss through sweat, although this is typically modest during short-duration exercise [46,47]. While CAF supplementation did not produce time-dependent Ca effects in the present study, chronic CAF consumption is known to increase urinary Ca excretion. Li and Koenig [11] reviewed evidence linking habitual coffee and CAF intake to increased urinary Ca losses, with potential implications for bone health in susceptible populations. However, acute CAF supplementation studies have yielded inconsistent findings regarding serum Ca dynamics [44,48]. These findings suggest that, within the context of a CrossFit® session preceded by a standardized meal, acute CAF supplementation (3–9 mg_CAF_/kg_BM_) does not significantly perturb serum Ca homeostasis beyond normal temporal fluctuations.

### 4.6. P: a unique medium-dose effect and exercise response

P concentration exhibited both significant ‘TR’ and ‘TP’ main effects, but no ‘TR’ × ‘TP’ interaction. Post hoc analysis for the ‘TR’ effect revealed that the *MEDIUM* CAF dose produced significantly higher P concentrations across all TP compared to *BASE*, while *LOW* and *HIGH* doses did not differ from *BASE* or *PLA*. For the ‘TP’ effect, post-exercise P concentrations at 90′ TP were markedly elevated compared with all earlier TP (0′, 30′, and 60′ TP), with a progressive increase from baseline to pre-exercise (60′ TP) and a further substantial post-exercise increase. This pattern is consistent with established exercise physiology, as the post-exercise elevation in serum P may result from phosphocreatine hydrolysis and adenosine triphosphate breakdown, cellular membrane permeability and muscle damage or acid-base shifts and P buffering. High-intensity muscle contraction rapidly depletes phosphocreatine and adenosine triphosphate, releasing inorganic P into the circulation [49]. Moreover, intense eccentric and concentric contractions during CrossFit® efforts may increase cellular permeability and potentially cause microtrauma, allowing intracellular P to leak into the bloodstream [50]. Additionally, exercise-induced acidosis alters the distribution of P between intracellular and extracellular compartments and influences protein binding of P [51]. On the other hand, the dose-specific elevation of P with *MEDIUM CAF* dose is intriguing and lacks clear precedent in the literature. One possible explanation may be the renal tubular effects, where *CAF* acts as a mild diuretic through adenosine receptor antagonism in the kidney, which may influence P excretion and reabsorption dynamics [52]. However, why this effect would be maximal at *MEDIUM* rather than *HIGH* dose is unclear, implying that the medium CAF dose effect on P concentration should be interpreted cautiously and requires replication.

### 4.7. Mg: homeostatic stability

Mg concentration exhibited no significant changes in response to ‘TR’, ‘TP’ or their interaction. This finding indicates robust homeostatic regulation of serum Mg during the CrossFit® workout with or without CAF supplementation. One of the factors explaining the lack of observable Mg changes may be the fact that serum Mg constitutes only ~1% of total body Mg, with the majority residing in bone (~60%) and intracellular compartments (~39%) [53]. Consequently, serum Mg concentrations are tightly regulated and may not reflect acute intracellular shifts or functional Mg status. Moreover, the moderately trained CrossFit® athletes likely had adequate Mg stores due to regular training adaptations and a habitual dietary intake that met the recommended dietary allowances, buffering against acute perturbations and the 17-min CrossFit® FGB workout may have been insufficient to significantly deplete Mg stores or overwhelm homeostatic regulatory mechanisms. The literature on CAF effects on Mg homeostasis is mixed. Some studies [54,55] report that CAF increases urinary Mg excretion and may transiently reduce serum or intracellular Mg concentrations, particularly with chronic high-dose consumption. These findings suggest that within the acute timeframe of a single CrossFit® session, CAF supplementation at doses up to 9 mg_CAF_/kg_BM_ does not significantly perturb serum Mg homeostasis in regularly trained athletes.

### 4.8. Integration of findings: selective CAF effects during the CrossFit® workout

Overall, the findings demonstrate that acute CrossFit® workout is the predominant driver of hematological changes, with CAF supplementation exerting highly selective effects on specific biochemical indices—most notably Glu and, to a lesser extent, P concentration. The absence of ‘TR’ × ‘TP’ interactions for other hematological variables and biochemical markers indicates that CAF does not significantly modify the acute physiological responses to high-intensity exercise within the examined range of doses (3–9 mg_CAF_/kg_BM_). This pattern contrasts with the well-established ergogenic effects of CAF on performance, perception of effort, reaction time, and postural stability documented in our previous work using the identical study protocol [16]. The limited effects observed for the analyzed hematological and biochemical markers, despite the previously reported performance-related outcomes from the same experimental trial, may indicate that these outcomes are not primarily explained by changes in the peripheral markers examined in the present study. However, the mechanisms underlying these effects were not directly investigated, and therefore no conclusions can be drawn regarding the contribution of central nervous system mechanisms.

### 4.9. Strengths

The present study has several notable strengths. First, the randomized, double-blind, *PLA*-controlled crossover design, involving different CAF doses and *BASE* visit, allowed for a robust assessment of the effects of three CAF doses, which is rarely explored in such detail in CrossFit® research. Second, the study was conducted on a well-defined group of 26 moderately trained CrossFit® athletes, a sample size that was *a priori* calculated and fully met, ensuring adequate statistical power. Third, we implemented a rigorous standardization protocol, including the same time of day for all visits to eliminate circadian rhythm influence, a 7-day washout period, and a standardized pre-visit meal to control for metabolic and glycemic baseline status. Furthermore, all participants were habitual CAF users who successfully maintained 24-h abstinence before each trial, confirmed by researchers. Finally, the application of the HCT conversion factor for all cellular and biochemical indices is a significant methodological advantage, as it allowed us to account for exercise-induced fluid shifts and avoid the misinterpretation of hematological and mineral concentration changes.

### 4.10. Limitations

Several limitations warrant acknowledgment. While the HCT conversion factor was applied to cellular components and biochemical indices to reduce the influence of exercise-induced hemoconcentration, plasma volume was not directly measured or corrected using validated methods (e.g. Dill and Costill calculations or carbon monoxide rebreathing). Furthermore, the use of a fixed reference hematocrit value (0.500 L·L^−1^) may not fully account for physiological differences in baseline hematocrit between male and female participants, and this should be considered when interpreting the corrected hematological variables. Therefore, it cannot be excluded that some of the observed hematological responses were influenced, at least in part, by exercise-induced plasma volume shifts rather than true cellular changes. This should be considered when interpreting the hematological findings. The study did not assess *CYP1A2* or *ADORA2A* polymorphisms, potentially obscuring subgroup-specific effects and contributing to unexplained inter-individual variability. Moreover, despite the high-intensity nature of the workout protocol, its relatively short duration may have been insufficient to elicit significant perturbations or to reveal certain physiological shifts that typically emerge during more prolonged exertion (e.g. Mg depletion, Ca losses through sweat). Additionally, the sensitivity of the mineral markers was limited, as serum Ca, Mg, and P concentrations often provide a limited reflection of total body status due to their strict homeostatic regulation. Moreover, although both male and female athletes were included, the study was not powered to detect sex-specific responses to CAF. Therefore, potential sex-related differences in hematological and biochemical responses cannot be excluded and should be investigated in future studies. In addition, the effectiveness of blinding was not formally evaluated. Therefore, particularly at the highest CAF dose (9 mg/kg_BM_), it cannot be excluded that some participants correctly identified the supplementation condition.

## 5. Conclusions

This study provides novel insights into the acute hematological and biochemical responses to acute CAF supplementation during the CrossFit® workout. The principal findings demonstrate that exercise is the predominant driver of hematological changes, with CAF exerting highly selective effects on post-exercise Glu responses. *MEDIUM* and *HIGH* CAF doses significantly increased post-exercise Glu concentrations compared to *BASE* and *PLA*, confirming the role of CAF as a metabolic modulator during high-intensity exercise. The *MEDIUM* dose also specifically elevated P concentrations, although the mechanism and functional significance remain unclear. Ca dynamics were influenced primarily by temporal factors related to exercise, while Mg remained stable across all conditions, indicating robust homeostatic regulation.

Critically, the observed ‘TR’ effects on HGB, HCT, and related RBC indices reflected differences between *BASE* visit and all subsequent experimental visits (including *PLA*), indicating methodological artifacts rather than CAF-specific pharmacological effects.

These findings indicate that acute CAF supplementation selectively influences post-exercise Glu responses during the CrossFit® workout while exerting minimal effects on hematological variables and most biochemical markers. These findings indicate that the analyzed hematological and biochemical markers are unlikely to fully explain the previously reported performance-related outcomes following acute caffeine supplementation. However, the mechanisms underlying these effects were not directly investigated in the present study. Future research incorporating genetic screening, plasma volume correction, mechanistic assessments, and extended time-course analyses will further elucidate the mechanisms and individual variability underlying these responses, enabling precision nutrition approaches for optimizing CAF supplementation in CrossFit® athletes.

## Supplementary Material

Supplementary material Table S2_r2.docx

Supplementary material Table S3_r2.docx

Supplementary material CONSORT.docx

Supplementary Material Table_S1_r2.docx

## Data Availability

The raw data supporting the conclusions of this article will be made available by the authors on request.
