# Supplementary material for: Hematological and biochemical responses to three doses of caffeine during the CrossFit® workout: a randomized, double-blind, crossover trial
Source: J Int Soc Sports Nutr. 2026 Oct 23;23(1):2740805. doi: 10.1080/15502783.2026.2740805 (PMC13700518; doi:10.1080/15502783.2026.2740805)
Supplement: Supplementary file 1 [file RSSN_A_2740805_SM5093.docx]

| Indicator | Treatment | Time point | | | | ‘TR’ mean: |
| --- | --- | --- | --- | --- | --- | --- |
|  |  | **1** | **2** | **3** | **4** |  |
| WBC  [10^9^∙L^-1^] | **B** | 7.62 (2.01) | 9.64 (2.82) | 10.82 (3.11) | 15.66 (3.66) | 10.93 |
|  | **L** | 7.61 (1.97) | 9.34 (2.21) | 10.12 (2.34) | 15.43 (3.12) | 10.62 |
|  | **M** | 7.71 (1.99) | 9.64 (2.59) | 10.35 (2.31) | 15.65 (4.09) | 10.84 |
|  | **H** | 7.64 (1.93) | 8.90 (2.64) | 10.77 (2.84) | 16.59 (3.88) | 10.98 |
|  | **PLA** | 7.71 (2.29) | 9.16 (2.40) | 9.82 (2.39) | 15.55 (3.52) | 10.56 |
|  | **‘TP’ mean:** | 7.66 | 9.34 | 10.38 | 15.77 |  |
|  |  | **1** | **2** | **3** | **4** | **‘TR’ mean:** |
| LYM  [10^9^∙L^-1^] | **B** | 3.12 (0.94) | 4.74 (1.54) | 5.35 (1.67) | 8.62 (1.94) | 5.46 |
|  | **L** | 3.13 (1.05) | 4.55 (1.21) | 5.11 (1.40) | 8.69 (1.88) | 5.37 |
|  | **M** | 3.23 (1.45) | 4.75 (1.58) | 5.28 (1.35) | 9.03 (2.14) | 5.57 |
|  | **H** | 3.23 (1.09) | 4.40 (1.29) | 5.67 (1.66) | 9.47 (1.75) | 5.69 |
|  | **PLA** | 3.07 (0.98) | 4.35 (1.35) | 4.47 (1.25) | 8.48 (1.93) | 5.16 |
|  | **‘TP’ mean:** | 3.16 | 4.56 | 5.23 | 8.86 |  |
|  |  | **1** | **2** | **3** | **4** | **‘TR’ mean:** |
| MON  [10^9^∙L^-1^] | **B** | 0.66 (0.18) | 0.75 (0.22) | 0.86 (0.25) | 1.23 (0.31) | 0.88 |
|  | **L** | 0.61 (0.18) | 0.73 (0.21) | 0.81 (0.23) | 1.17 (0.29) | 0.83 |
|  | **M** | 0.60 (0.18) | 0.74 (0.23) | 0.80 (0.24) | 1.22 (0.40) | 0.84 |
|  | **H** | 0.58 (0.17) | 0.68 (0.16) | 0.82 (0.21) | 1.28 (0.30) | 0.82 |
|  | **PLA** | 0.60 (0.16) | 0.69 (0.17) | 0.76 (0.20) | 1.22 (0.32) |  |
|  | **‘TP’ mean:** | 0.61 | 0.72 | 0.81 | 1.22 |  |
|  |  | **1** | **2** | **3** | **4** | **‘TR’ mean:** |
| GRA  [10^9^∙L^-1^] | **B** | 3.85 (1.33) | 4.15 (1.52) | 4.62 (1.68) | 5.80 (2.09) | 4.61 |
|  | **L** | 3.87 (1.22) | 4.07 (1.30) | 4.23 (1.38) | 5.55 (1.65) | 4.43 |
|  | **M** | 3.85 (1.02) | 4.14 (1.23) | 4.28 (1.07) | 5.34 (1.84) | 4.40 |
|  | **H** | 3.81 (1.38) | 3.83 (1.51) | 4.29 (1.57) | 5.84 (2.53) | 4.44 |
|  | **PLA** | 4.03 (1.44) | 4.11 (1.32) | 4.34 (1.30) | 5.86 (1.62) | 4.58 |
|  | **‘TP’ mean:** | 3.88 | 4.06 | 4.35 | 5.68 |  |

**Table S1.** Means with standard deviations (SD) of hematological variables.

**Table S1. Continued.**

| Indicator | Treatment | Time Point | | | | ‘TR’ mean: |
| --- | --- | --- | --- | --- | --- | --- |
|  |  | **1** | **2** | **3** | **4** |  |
| LYM  [%] | **B** | 40.98 (7.01) | 49.23 (7.33) | 49.57 (7.31) | 55.72 (6.41) | 48.88 |
|  | **L** | 41.10 (8.56) | 48.98 (7.53) | 50.53 (7.82) | 56.54 (6.64) | 49.29 |
|  | **M** | 41.28 (9.12) | 49.13 (7.38) | 50.88 (6.11) | 58.72 (7.37) | 50.00 |
|  | **H** | 42.63 (9.02) | 49.69 (6.52) | 52.70 (8.01) | 57.97 (7.63) | 50.75 |
|  | **PLA** | 40.29 (6.58) | 47.66 (7.39) | 48.33(6.48) | 54.52 (6.32) | 47.70 |
|  | **‘TP’ mean:** | 41.26 | 48.94 | 50.40 | 56.69 |  |
|  |  | **1** | **2** | **3** | **4** | **‘TR’ mean:** |
| MON  [%] | **B** | 9.01 (2.82) | 7.99 (1.41) | 8.13 (1.59) | 7.95 (1.38) | 8.27 |
|  | **L** | 8.02 (2.35) | 7.72 (1.50) | 7.97 (2.23) | 7.66 (1.12) | 7.84 |
|  | **M** | 8.02 (1.96) | 7.82 (1.09) | 7.61 (0.99) | 7.62 (1.10) | 7.77 |
|  | **H** | 7.99 (2.38) | 7.89 (1.25) | 7.61 (0.99) | 7.76 (1.07) | 7.81 |
|  | **PLA** | 7.92 (1.77) | 7.60 (1.03) | 7.70 (1.47) | 7.82 (0.97) | 7.76 |
|  | **‘TP’ mean:** | 8.19 | 7.80 | 7.80 | 7.76 |  |
|  |  | **1** | **2** | **3** | **4** | **‘T’ av.:** |
| GRA  [%] | **B** | 50.01 (7.13) | 42.78 (7.76) | 42.30 (7.69) | 36.33 (6.92) | 42.86 |
|  | **L** | 50.88 (8.97) | 43.30 (8.07) | 41.50 (8.61) | 35.80 (6.84) | 42.87 |
|  | **M** | 50.70 (9.18) | 43.05 (7.61) | 41.51 (6.45) | 33.67 (7.05) | 42.23 |
|  | **H** | 49.37 (9.71) | 42.42 (7.12) | 39.70 (8.49) | 34.27 (7.83) | 41.44 |
|  | **PLA** | 51.79 (6.51) | 44.74 (7.35) | 43.96 (6.44) | 37.66 (6.32) | 44.54 |
|  | **‘TP’ mean:** | 50.55 | 43.26 | 41.80 | 35.55 |  |
|  |  | **1** | **2** | **3** | **4** | **‘TR’ mean:** |
| RBC  [10^12^∙L^-1^] | **B** | 5.66 (0.33) | 5.66 (0.33) | 5.66 (0.32) | 5.59 (0.33) | 5.64 |
|  | **L** | 5.69 (0.32) | 5.70 (0.31) | 5.69 (0.32) | 5.60 (0.31) | 5.67 |
|  | **M** | 5.70 (0.30) | 5.71 (0.28) | 5.70 (0.29) | 5.63 (0.30) | 5.68 |
|  | **H** | 5.69 (0.28) | 5.72 (0.30) | 5.70 (0.28) | 5.61 (0.28) | 5.68 |
|  | **PLA** | 5.68 (0.29) | 5.72 (0.25) | 5.70 (0.28) | 5.62 (0.29) | 5.68 |
|  | **‘TP’ mean:** | 5.68 | 5.70 | 5.69 | 5.61 |  |

**Table S1. Continued.**

| Indicator | Treatment | Time point | | | | ‘TR’ mean: |
| --- | --- | --- | --- | --- | --- | --- |
|  |  | **1** | **2** | **3** | **4** |  |
| HGB  [mmol∙L^-1^] | **B** | 10.21 (0.50) | 10.06 (0.39) | 9.99 (0.37) | 9.82 (0.46) | 10.02 |
|  | **L** | 10.49 (0.46) | 10.34 (0.35) | 10.33 (0.31) | 10.09 (0.31) | 10.31 |
|  | **M** | 10.55 (0.39) | 10.39 (0.32) | 10.35 (0.26) | 10.07 (0.44) | 10.34 |
|  | **H** | 10.49 (0.47) | 10.35 (0.29) | 10.27 (0.38) | 10.03 (0.42) | 10.29 |
|  | **PLA** | 10.50 (0.45) | 10.36 (0.48) | 10.30 (0.56) | 10.10(0.42) | 10.31 |
|  | **‘TP’ mean:** | 10.45 | 10.30 | 10.25 | 10.02 |  |
|  |  | **1** | **2** | **3** | **4** | **‘TR’ mean:** |
| HTC  [L∙L^-1^] | **B** | 0.42 (0.03) | 0.42 (0.03) | 0.43 (0.04) | 0.44 (0.04) | 0.43 |
|  | **L** | 0.42 (0.03) | 0.42 (0.03) | 0.42 (0.04) | 0.44 (0.04) | 0.42 |
|  | **M** | 0.42 (0.03) | 0.41 (0.04) | 0.42 (0.04) | 0.43 (0.06) | 0.42 |
|  | **H** | 0.42 (0.03) | 0.41 (0.03) | 0.42 (0.03) | 0.45 (0.04) | 0.42 |
|  | **PLA** | 0.41 (0.03) | 0.40 (0.03) | 0.41 (0.04) | 0.44 (0.03) | 0.41 |
|  | **‘TP’ mean:** | 0.42 | 0.41 | 0.42 | 0.44 |  |
|  |  | **1** | **2** | **3** | **4** | **‘TR’ mean:** |
| MCV  [fL] | **B** | 88.65 (5.35) | 88.57 (5.24) | 88.64 (5.18) | 89.86 (5.47) | 88.91 |
|  | **L** | 88.19 (5.11) | 88.00 (5.07) | 88.14 (5.10) | 89.60 (5.17) | 88.48 |
|  | **M** | 88.00 (4.72) | 87.76 (4.48) | 87.87 (4.64) | 89.04 (4.92) | 88.17 |
|  | **H** | 88.06 (4.57) | 87.70 (4.77) | 87.95 (4.50) | 89.31 (4.73) | 88.25 |
|  | **PLA** | 88.25 (4.60) | 88.02 (4.54) | 88.00 (4.45) | 89.23 (4.78) | 88.38 |
|  | **‘TP’ mean:** | 88.23 | 88.01 | 88.12 | 89.39 |  |
|  |  | **1** | **2** | **3** | **4** | **‘TR’ mean:** |
| MCH  [fmol] | **B** | 1.81 (0.11) | 1.78 (0.12) | 1.77 (0.11) | 1.76 (0.11) | 1.78 |
|  | **L** | 1.85 (0.12) | 1.82 (0.12) | 1.82 (0.13) | 1.81 (0.12) | 1.82 |
|  | **M** | 1.86 (0.13) | 1.83 (0.12) | 1.81 (0.12) | 1.79 (0.13) | 1.82 |
|  | **H** | 1.85 (0.14) | 1.82 (0.11) | 1.81 (0.13) | 1.79 (0.13) | 1.82 |
|  | **PLA** | 1.85 (0.14) | 1.82 (0.14) | 1.81 (0.14) | 1.80 (0.13) | 1.82 |
|  | **‘TP’ mean:** | 1.84 | 1.81 | 1.80 | 1.79 |  |

**Table S1. Continued.**

| Indicator | Treatment | Time point | | | | ‘TR’ mean: |
| --- | --- | --- | --- | --- | --- | --- |
|  |  | **1** | **2** | **3** | **4** |  |
| MCHC  [mmol∙L^-1^] | **B** | 20.43 (1.00) | 20.13 (0.78) | 19.98 (0.76) | 19.63 (0.92) | 20.04 |
|  | **L** | 20.97 (0.92) | 20.69 (0.69) | 20.67 (0.62) | 20.18 (0.62) | 20.63 |
|  | **M** | 21.09 (0.78) | 20.78 (0.65) | 20.70 (0.42) | 20.12 (0.85) | 20.67 |
|  | **H** | 21.00 (0.94) | 20.71 (0.58) | 20.54 (0.75) | 20.07 (0.84) | 20.58 |
|  | **PLA** | 21.00 (0.90) | 20.68 (0.89) | 20.60 (1.11) | 20.19 (0.85) | 20.62 |
|  | **‘TP’ mean:** | 20.90 | 20.60 | 20.50 | 20.04 |  |
|  |  | **1** | **2** | **3** | **4** | **‘TR’ mean:** |
| RDW-C  [%] | **B** | 12.54 (0.92) | 12.68 (0.93) | 12.84 (0.90) | 12.68 (0.90) | 12.69 |
|  | **L** | 12.68 (1.05) | 12.81 (1.08) | 12.83 (1.12) | 12.68 (1.18) | 12.75 |
|  | **M** | 12.66 (1.04) | 12.60 (1.01) | 12.86 (0.95) | 12.65 (1.08) | 12.69 |
|  | **H** | 12.52 (1.08) | 12.67 (0.98) | 12.70 (1.14) | 12.72 (1.17) | 12.65 |
|  | **PLA** | 12.85 (1.26) | 12.79 (1.22) | 12.85 (1.09) | 12.71 (1.25) | 12.80 |
|  | **‘TP’ mean:** | 12.65 | 12.71 | 12.82 | 12.69 |  |
|  |  | **1** | **2** | **3** | **4** | **‘TR’ mean:** |
| RDW-S  [fL] | **B** | 43.43 (3.15) | 43.85 (3.67) | 43.98 (3.46) | 35.16 (4.16) | 44.12 |
|  | **L** | 43.31 (3.19) | 43.61 (3.74) | 43.73 (3.57) | 43.72 (2.99) | 43.59 |
|  | **M** | 42.79 (2.98) | 43.43 (2.94) | 43.79 (2.76) | 44.49 (3.45) | 43.63 |
|  | **H** | 43.21 (3.12) | 43.14 (2.88) | 43.09 (3.16) | 44.22 (3.82) | 43.42 |
|  | **PLA** | 43.35 (2.62) | 43.14 (2.57) | 43.21 (2.82) | 43.56 (2.10) | 43.31 |
|  | **‘TP’ mean:** | 43.22 | 43.44 | 43.56 | 44.24 |  |
|  |  | **1** | **2** | **3** | **4** | **‘TR’ mean:** |
| PLT  [10^9^∙L^-1^] | **B** | 281.08 (103.71) | 210.03 (81.77) | 206.03 (91.62) | 184.16 (95.62) | 220.32 |
|  | **L** | 276.00 (79.29) | 191.02 (77.68) | 205.47 (101.01) | 212.41 (112.39) | 221.23 |
|  | **M** | 287.47 (101.28) | 221.28 (89.46) | 213.35 (91.03) | 191.07 (111.94) | 228.29 |
|  | **H** | 284.37 (97.96) | 246.38 (110.45) | 264.91 (89.47) | 195.68 (79.27) | 247.83 |
|  | **PLA** | 292.28 (67.44) | 221.35 (90.04) | 250.91 (87.58) | 207.92 (84.40) | 243.12 |
|  | **‘TP’ mean:** | 284.24 | 218.01 | 228.13 | 198.25 |  |

**Table S1. Continued.**

| Indicator | Treatment | Time point | | | | ‘TR’ mean: |
| --- | --- | --- | --- | --- | --- | --- |
|  |  | **1** | **2** | **3** | **4** |  |
| MPV  [fL] | **B** | 8.17 (0.75) | 8.29 (0.67) | 8.40 (0.58) | 8.74 (0.72) | 8.40 |
|  | **L** | 8.10 (0.91) | 8.49 (0.81) | 8.29 (0.75) | 8.61 (0.77) | 8.37 |
|  | **M** | 8.22 (0.74) | 8.32 (0.70) | 8.28 (0.50) | 8.80 (0.69) | 8.40 |
|  | **H** | 8.13 (0.78) | 8.31 (0.70) | 8.37 (0.71) | 8.85 (0.57) | 8.41 |
|  | **PLA** | 8.15 (0.86) | 8.26 (0.66) | 8.32 (0.62) | 8.70 (0.68) | 8.36 |
|  | **‘TP’ mean:** | 8.15 | 8.34 | 8.33 | 8.74 |  |
|  |  | **1** | **2** | **3** | **4** | **‘TR’ mean:** |
| PCT  [cl∙L^-1^] | **B** | 0.19 (0.06) | 0.14 (0.05) | 0.15 (0.06) | 0.14 (0.06) | 0.16 |
|  | **L** | 0.18 (0.05) | 0.13 (0.05) | 0.14 (0.06) | 0.16 (0.08) | 0.15 |
|  | **M** | 0.19 (0.06) | 0.15 (0.05) | 0.15 (0.06) | 0.14 (0.08) | 0.16 |
|  | **H** | 0.19 (0.06) | 0.15 (0.08) | 0.16 (0.06) | 0.15 (0.06) | 0.16 |
|  | **PLA** | 0.19 (0.04) | 0.14 (0.06) | 0.16 (0.06) | 0.15 (0.06) | 0.16 |
|  | **‘TP’ mean:** | 0.19 | 0.15 | 0.15 | 0.15 |  |
|  |  | **1** | **2** | **3** | **4** | **‘TR’ mean:** |
| PDW  [%] | **B** | 12.77 (1.04) | 12.44 (1.13) | 11.81 (1.88) | 11.89 (1.92) | 12.23 |
|  | **L** | 12.62 (1.41) | 11.76 (1.57) | 11.71 (1.92) | 11.60 (1.77) | 11.92 |
|  | **M** | 12.42 (1.03) | 11.62 (1.49) | 11.87 (1.28) | 11.49 (2.20) | 11.85 |
|  | **H** | 12.71 (1.30) | 11.67 (1.58) | 12.16 (1.20) | 11.83 (1.33) | 12.09 |
|  | **PLA** | 12.53 (1.39) | 11.97 (1.43) | 12.17 (1.55) | 11.93 (1.67) | 12.15 |
|  | **‘TP’ mean:** | 12.61 | 11.89 | 11.94 | 11.75 |  |
|  |  | **1** | **2** | **3** | **4** | **‘TR’ mean:** |
| PLCR  [%] | **B** | 19.03 (8.21) | 22.80 (8.38) | 23.07 (9.24) | 25.47 (10.03) | 22.59 |
|  | **L** | 19.03 (8.40) | 23.47 (8.19) | 21.41 (8.98) | 24.58 (8.70) | 22.12 |
|  | **M** | 20.98 (9.49) | 21.06 (6.16) | 22.47 (8.21) | 28.19 (11.01) | 23.18 |
|  | **H** | 18.91 (7.90) | 22.30 (8.62) | 22.58 (8.23) | 28.00 (10.24) | 22.95 |
|  | **PLA** | 19.60 (8.99) | 21.52 (8.34) | 21.93 (8.34) | 26.38 (9.28) | 22.36 |
|  | **‘TP’ mean:** | 19.51 | 22.23 | 22.29 | 26.53 |  |

WBC – white blood cells; LYM – lymphocytes; MON – monocytes; GRA – granulocytes; RBC – red blood cells count; HGB – hemoglobin concentration; HCT – hematocrit; MCH – mean corpuscular hemoglobin mass; MCHC – mean corpuscular hemoglobin concentration; MCV – mean corpuscular volume; MPV – mean platelet volume; PCT – platelet hematocrit; PDW – platelet distribution width; PLT – platelet count; PLCR – platelet large cell ratio; ‘TR’ – treatment; ‘TP’ – time point; B – baseline; L – 3 mg/kg_BM_ acute caffeine dose; M - 6 mg/kg_BM_ acute caffeine dose; H - 9 mg/kg_BM_ acute caffeine dose; PLA – placebo; 1-4 – blood sample collection [1] pre-supplementation, [2] 30 minutes after supplementation, [3] 60 minutes after supplementation, [4] 3 minutes post-exercise.
