# Supplementary material for: Hematological and biochemical responses to three doses of caffeine during the CrossFit® workout: a randomized, double-blind, crossover trial
Source: J Int Soc Sports Nutr. 2026 Oct 23;23(1):2740805. doi: 10.1080/15502783.2026.2740805 (PMC13700518; doi:10.1080/15502783.2026.2740805)
Supplement: Supplementary file 2 [file RSSN_A_2740805_SM5094.docx]

**Table S2.** Distribution of potentially outlying observations identified using the 1.5 × IQR criterion

| **Variable** | **IQR-flagged observations**  **n** | **IQR-flagged observations**  **%** |
| --- | --- | --- |
| WBC | 5 | 0.96% |
| LYM | 7 | 1.35% |
| MON | 2 | 0.38% |
| GRA | 5 | 0.96% |
| LYM% | 0 | 0.00% |
| MON% | 3 | 0.58% |
| GRA% | 1 | 0.19% |
| RBC | 1 | 0.19% |
| HGB | 5 | 0.96% |
| HCT | 2 | 0.38% |
| MCV | 2 | 0.38% |
| MCH | 4 | 0.77% |
| MCHC | 1 | 0.19% |
| RDW-C | 0 | 0.00% |
| RDW-S | 3 | 0.58% |
| PLT | 0 | 0.00% |
| MVP | 0 | 0.00% |
| PCT | 1 | 0.19% |
| PDW | 1 | 0.19% |
| PLCR | 1 | 0.19% |
| Glucose* | 22 / 8 | 4.23% / 1.60% |
| Calcium | 5 | 0.96% |
| Magnesium | 2 | 0.38% |
| Phosphates | 7 | 1.35% |
| **Total** | **80** | **0.64%** |

* values presented before / after exclusion of one participant from glucose-specific analysis
