# Supplementary material for: Hematological and biochemical responses to three doses of caffeine during the CrossFit® workout: a randomized, double-blind, crossover trial
Source: J Int Soc Sports Nutr. 2026 Oct 23;23(1):2740805. doi: 10.1080/15502783.2026.2740805 (PMC13700518; doi:10.1080/15502783.2026.2740805)
Supplement: Supplementary file 3 [file RSSN_A_2740805_SM5095.docx]

**Table S3.** Comparison of omnibus effects between the primary analysis including the *BASE* condition and the sensitivity analysis excluding the *BASE* condition

| **Variable** | **‘TR’ × ‘TP’ Primary** | **‘TR’ × ‘TP’ Sensitivity** | **‘TR’ Primary** | **‘TR’ Sensitivity** | **‘TP’ Primary** | **‘TP’**  **Sensitivity** |
| --- | --- | --- | --- | --- | --- | --- |
| **WBC** | *F =* 1.43  *p* = 0.224  *η_p_²* = 0.05 | *F =* 1.53  *p* = 0.206  *η_p_²* = 0.06 | *F =* 0.50  *p* = 0.665  *η_p_²* = 0.02 | *F =* 0.54  *p* = 0.601  *η_p_²* = 0.02 | *F =* 280.38  *p* < 0.001  *η_p_²* = 0.92 | *F =* 241.00  *p* < 0.001  *η_p_²* = 0.91 |
| **LYM** | *F =* 1.57  *p* = 0.149  *η_p_²* = 0.06 | *F =* 1.69  *p* = 0.142  *η_p_²* = 0.06 | *F = 2*.32  *p* = 0.081  *η_p_²* = 0.08 | *F =* 2.97  *p* = 0.052  *η_p_²* = 0.11 | *F =* 328.29  *p <* 0.001  *η_p_²* = 0.93 | *F =* 284.00  *p* < 0.001  *η_p_²* = 0.92 |
| **MON** | *F =* 1.04  *p* = 0.397  *η_p_²* = 0.04 | *F =* 1.09  *p* = 0.362  *η_p_²* = 0.04 | *F =* 0.95  *p* = 0.440  *η_p_²* = 0.04 | *F =* 0.24  *p* = 0.868  *η_p_²* = 0.01 | *F =* 137.82  *p* < 0.001  *η_p_²* = 0.85 | *F =* 138.00  *p* < 0.001  *η_p_²* = 0.85 |
| **GRA** | *F =* 1.53  *p* = 0.212  *η_p_²* = 0.06 | *F =* 1.47  *p* = 0.234  *η_p_²* = 0.06 | *F =* 0.30  *p* = 0.874  *η_p_²* = 0.01 | *F =* 0.23  *p* = 0.820  *η_p_²* = 0.01 | *F =* 106.20  *p* < 0.001  *η_p_²* = 0.81 | *F =* 89.30  *p* < 0.01  *η_p_²* = 0.78 |
| **LYM%** | *F =* 0.87  *p* = 0.524  *η_p_²* = 0.03 | *F =* 0.89  *p* = 0.490  *η_p_²* = 0.03 | *F = 2*.22  *p* = 0.072  *η_p_²* = 0.08 | ***F = 2*.75**  ***p* = 0.048**  ***η_p_²* = 0.10** | *F =* 181.41  *p* < 0.001  *η_p_²* = 0.88 | *F =* 147.00  *p* < 0.001  *η_p_²* = 0.85 |
| **MON%** | *F =* 1.54  *p* = 0.166  *η_p_²* = 0.06 | *F =* 0.67  *p* = 0.661  *η_p_²* = 0.03 | *F =* 0.90  *p* = 0.432  *η_p_²* = 0.03 | *F =* 0.03  *p* = 0.973  *η_p_²* < 0.01 | *F = 2*.34  *p* = 0.126  *η_p_²* = 0.09 | *F =* 0.93  *p* = 0.380  *η_p_²* = 0.04 |
| **GRA%** | *F =* 0.89  *p* = 0.506  *η_p_²* = 0.03 | *F =* 0.79  *p* = 0.563  *η_p_²* = 0.03 | *F =* 1.83  *p* = 0.129  *η_p_²* = 0.07 | *F =* 2.42  *p* = 0.073  *η_p_²* = 0.09 | *F =* 177.99  *p* < 0.001  *η_p_²* = 0.88 | *F =* 147.00  *p* < 0.001  *η_p_²* = 0.85 |
| **RBC** | *F =* 0.53  *p* = 0.672  *η_p_²* = 0.02 | *F =* 0.41  *p* = 0.730  *η_p_²* = 0.02 | *F =* 0.62  *p* = 0.569  *η_p_²* = 0.03 | *F =* 0.16  *p* = 0.881  *η_p_²* = 0.01 | *F =* 52.18  *p* < 0.001  *η_p_²* = 0.68 | *F =* 37.90  *p* < 0.001  *η_p_²* = 0.60 |
| **HGB** | *F =* 0.33  *p* = 0.922  *η_p_²* = 0.01 | *F =* 0.37  *p* = 0.874  *η_p_²* = 0.02 | *F =* 6.57  *p* < 0.001  *η_p_²* = 0.21 | ***F =* 0.08**  ***p* = 0.968**  ***η_p_²* < 0.01** | *F =* 58.20  *p* < 0.001  *η_p_²* = 0.70 | *F =* 53.30  *p* < 0.001  *η_p_²* = 0.68 |
| **HCT** | *F =* 1.96  *p* = 0.068  *η_p_²* = 0.07 | *F =* 2.00  *p* = 0.086  *η_p_²* = 0.07 | *F =* 2.71  *p* = 0.035  *η_p_²* = 0.10 | ***F =* 1.74**  ***p* = 0.166**  ***η_p_²* = 0.07** | *F =* 37.55  *p* < 0.001  *η_p_²* = 0.60 | *F =* 38.70  *p* < 0.001  *η_p_²* = 0.61 |
| **MCV** | *F =* 0.57  *p* = 0.620  *η_p_²* = 0.02 | *F =* 0.58  *p* = 0.589  *η_p_²* = 0.02 | *F =* 0.75  *p* = 0.506  *η_p_²* = 0.03 | *F =* 0.28  *p* = 0.769  *η_p_²* = 0.01 | *F =* 71.45  *p* < 0.001  *η_p_²* = 0.74 | *F =* 51.20  *p* < 0.001  *η_p_²* = 0.67 |
| **MCH** | *F =* 0.39  *p* = 0.883  *η_p_²* = 0.02 | *F =* 0.50  *p* = 0.772  *η_p_²* = 0.02 | *F =* 3.21  *p* = 0.031  *η_p_²* = 0.11 | ***F =* 0.17**  ***p* = 0.918**  ***η_p_²* = 0.01** | *F =* 35.28  *p* < 0.001  *η_p_²* = 0.59 | *F =* 30.20  *p* < 0.001  *η_p_²* = 0.55 |
| **MCHC** | *F =* 0.40  *p* = 0.884  *η_p_²* = 0.02 | *F =* 0.46  *p* = 0.810  *η_p_²* = 0.02 | *F =* 6.54  *p* < 0.001  *η_p_²* = 0.21 | ***F =* 0.07**  ***p* = 0.977**  ***η_p_²* < 0.01** | *F =* 57.83  *p* < 0.001  *η_p_²* = 0.70 | *F =* 52.70  *p* < 0.001  *η_p_²* = 0.68 |
| **RDW-C** | *F =* 1.00  *p* = 0.447  *η_p_²* = 0.04 | *F =* 1.03  *p* = 0.421  *η_p_²* = 0.04 | *F =* 0.40  *p* = 0.808  *η_p_²* = 0.02 | *F =* 0.45  *p* = 0.720  *η_p_²* = 0.02 | *F =* 4.17  *p* = 0.019  *η_p_²* = 0.14 | ***F =* 2.15**  ***p* = 0.101**  ***η_p_²* = 0.08** |
| **RDW-S** | *F =* 1.75  *p* = 0.132  *η_p_²* = 0.07 | *F =* 1.83  *p* = 0.144  *η_p_²* = 0.07 | *F =* 1.53  *p* = 0.198  *η_p_²* = 0.06 | *F =* 0.47  *p* = 0.705  *η_p_²* = 0.02 | *F =* 16.46  *p* < 0.001  *η_p_²* = 0.40 | *F =* 7.54  *p* < 0.001  *η_p_²* = 0.23 |
| **PLT** | *F =* 1.53  *p* = 0.112  *η_p_²* = 0.06 | *F =* 1.84  *p* = 0.062  *η_p_²* = 0.07 | *F =* 2.47  *p* = 0.049  *η_p_²* = 0.09 | *F =* 2.74  *p* = 0.049  *η_p_²* = 0.10 | *F =* 34.53  *p* < 0.001  *η_p_²* = 0.58 | *F =* 24.10  *p* < 0.001  *η_p_²* = 0.49 |
| **MPV** | *F =* 1.05  *p* = 0.405  *η_p_²* = 0.04 | *F =* 1.30  *p* = 0.239  *η_p_²* = 0.05 | *F =*0.26  *p* = 0.852  *η_p_²* = 0.01 | *F =* 0.39  *p* = 0.764  *η_p_²* = 0.02 | *F =* 24.91  *p <* 0.001  *η_p_²* = 0.50 | *F =* 21.80  *p* < 0.001  *η_p_²* = 0.47 |
| **PCT** | *F =* 0.70  *p* = 0.753  *η_p_²* = 0.03 | *F =* 0.89  *p* = 0.533  *η_p_²* = 0.04 | *F =* 0.48  *p* = 0.753  *η_p_²* = 0.02 | *F =* 0.54  *p* = 0.659  *η_p_²* = 0.02 | *F =* 22.39  *p* < 0.001  *η_p_²* = 0.47 | *F =* 15.00  *p* < 0.001  *η_p_²* = 0.38 |
| **PDW** | *F =* 0.45  *p* = 0.875  *η_p_²* = 0.02 | *F =* 0.22  *p* = 0.964  *η_p_²* = 0.01 | *F =* 1.40  *p* = 0.239  *η_p_²* = 0.05 | *F =* 1.21  *p* = 0.312  *η_p_²* = 0.05 | *F = 9*.05  *p* < 0.001  *η_p_²* = 0.27 | *F =* 6.99  *p* < 0.001  *η_p_²* = 0.22 |
| **PLCR** | *F =* 1.84  *p* = 0.087  *η_p_²* = 0.07 | *F =* 1.83  *p* = 0.101  *η_p_²* = 0.07 | *F =* 0.85  *p* = 0.499  *η_p_²* = 0.03 | *F =* 1.23  *p* = 0.303  *η_p_²* = 0.05 | *F =* 36.84  *p* < 0.001  *η_p_²* = 0.60 | *F =* 33.50  *p* < 0.001  *η_p_²* = 0.57 |
| **Glucose** | *F =* 3.25  *p* = 0.006  *η_p_²* = 0.12 | *F =* 3.70  *p* = 0.005  *η_p_²* = 0.13 | *F =* 4.23  *p* = 0.003  *η_p_²* = 0.15 | ***F =* 1.47**  ***p* = 0.231**  ***η_p_²* = 0.06** | *F =* 55.08  *p* < 0.001  *η_p_²* = 0.70 | *F =* 56.70  *p* < 0.001  *η_p_²* = 0.70 |
| **Calcium** | *F =* 0.64  *p* = 0.810  *η_p_²* = 0.02 | *F =* 0.75  *p* = 0.661  *η_p_²* = 0.029 | *F =* 0.50  *p* = 0.739  *η_p_²* = 0.02 | *F =* 0.33  *p* = 0.803  *η_p_²* = 0.01 | *F =* 11.91  *p* < 0.001  *η_p_²* = 0.32 | *F =* 12.30  *p* < 0.001  *η_p_²* = 0.33 |
| **Magnesium** | *F =* 1.49  *p* = 0.127  *η_p_²* = 0.06 | *F =* 1.97  *p* = 0.094  *η_p_²* = 0.07 | *F = 1*.30  *p* = 0.277  *η_p_²* = 0.05 | *F =* 1.64  *p* = 0.187  *η_p_²* = 0.06 | *F =* 1.11  *p* = 0.351  *η_p_²* = 0.04 | *F =* 0.56  *p* = 0.599  *η_p_²* = 0.02 |
| **Phosphates** | *F =* 1.44  *p* = 0.146  *η_p_²* = 0.05 | *F =* 1.67  *p* = 0.136  *η_p_²* = 0.063 | *F =* 2.95  *p* = 0.024  *η_p_²* = 0.11 | ***F =* 2.34**  ***p* = 0.080**  ***η_p_²* = 0.086** | *F =* 179.15  *p* < 0.001  *η_p_²* = 0.88 | *F =* 166.00  *p* < 0.001  *η_p_²* = 0.87 |

WBC – white blood cells; LYM – lymphocytes; MON – monocytes; GRA – granulocytes; RBC – red blood cells count; HGB – hemoglobin concentration; HCT – hematocrit; MCV – mean corpuscular volume; MCH – mean corpuscular hemoglobin mass; MCHC – mean corpuscular hemoglobin concentration; RDW-C – red cell distribution width – coefficient of variation; RDW-S – red cell distribution width – standard deviation; MPV – mean platelet volume; PCT – platelet hematocrit; PDW – platelet distribution width; PLT – platelet count; PLCR – platelet large cell ratio; ‘TR’×’TP’ – interaction effect of treatment and time point; ‘TR’ – main effect of treatment; ‘TP’ – main effect of time point; *F* – test statistic; *η_p_^2^* – partial eta squared.
